# Comparative efficacy and safety of medical treatments for proximal humerus fractures: a systematic review and network meta-analysis

**DOI:** 10.1186/s12891-023-07053-x

**Published:** 2024-01-02

**Authors:** Yun Zheng, Nan Tang, Wen-Jie Zhang, Wei Shi, Wen-Wen Zhao, Kun Yang

**Affiliations:** 1 R & D Center, Beijing Naton Technology Group Co., Ltd, Peking, China; 2 Director of Tianjin Center for Medical Devices Evaluation and Inspection, Tianjin, China; 3 Comprehensive Business Department, Tianjin Center for Medical Devices Evaluation and Inspection, Tianjin, China; 4 Quality Management Department, Tianjin Center for Medical Devices Evaluation and Inspection, Tianjin, China; 5 Registration and Regulatory Department, Beijing Naton Technology Group Co, Ltd, Building 1, Yard 9, Chengwan Street, Haidian District, Peking, 100094 China

**Keywords:** Open reduction internal fixation, Intramedullary nailing, Hemiarthroplasty, Reverse total shoulder arthroplasty, Proximal humerus fracture, Meta-analysis

## Abstract

**Background:**

Various surgical techniques and conservative therapies are useful tools for treating proximal humerus fractures (PHFs), but it is important to understand how to properly utilize them. Therefore, we performed a systematic review and network meta-analysis to compare and rank the efficacy and safety of medical treatments for PHF.

**Methods:**

PubMed, Embase, the Cochrane Library, and the ClinicalTrials.gov databases were systematically searched for eligible randomized controlled trials (RCTs) from inception until June 2022. Conservative therapy-controlled or head-to-head RCTs of open reduction internal fixation (ORIF), intramedullary nailing (IMN), hemiarthroplasty (HA), and reverse total shoulder arthroplasty (RTSA) used for the treatment of adult patients with PHF were included. The surface under the cumulative ranking (SUCRA) probabilities were applied to compare and rank the effects of medical treatments for PHF.

**Results:**

Eighteen RCTs involving 1,182 patients with PHF were selected for the final analysis. Mostly baseline characteristics among groups were well balanced, and the imbalanced factors only included age, injury type, medial comminution, blood loss, and cognitive function in single trial. The SUCRA probabilities found that RTSA provided the best effect on the Constant-Murley score (SUCRA: 100.0%), and the disabilities of the arm, shoulder and hand (DASH) score (SUCRA: 99.0%). Moreover, HA (SUCRA: 85.5%) and RTSA (SUCRA: 68.0%) had a relatively better effect on health-related quality of life than the other treatment modalities. Furthermore, conservative therapy (SUCRA: 84.3%) and RTSA (SUCRA: 80.7%) were associated with a lower risk of secondary surgery. Finally, the best effects on the risk of complications are varied, including infection was observed with conservative therapy (SUCRA: 94.2%); avascular necrosis was observed in HA (SUCRA: 78.1%), nonunion was observed in RTSA (SUCRA: 69.6%), and osteoarthritis was observed in HA (SUCRA: 93.9%).

**Conclusions:**

This study found that RTSA was associated with better functional outcomes, while the comparative outcomes of secondary surgery and complications varied. Optimal treatment for PHF should consider patient-specific factors.

**Supplementary Information:**

The online version contains supplementary material available at 10.1186/s12891-023-07053-x.

## Background

Proximal humerus fractures (PHFs) are the most frequent bone fractures, accounting for 5.7% of all fractures in the elderly, and are considered the third most common fracture after femur and wrist fractures [1, 2]. Given the aging of the population, the prevalence of PHFs is increasing constantly [3, 4]. This could be explained by the unimodal distribution of PHFs, peaking in the aged, and by the fact that PHFs are regarded as typical of osteoporotic injuries [5]. A patient’s age, bone quality, comorbidities, compliance, and functional demands could affect choice of treatment strategies for PHFs [6]. Most patients are treated with a nonsurgical approach, and sling immobilization is widely used for PHFs [7]. Nevertheless, surgical treatments such as open reduction internal fixation (ORIF), intramedullary nailing (IMN), hemiarthroplasty (HA), and reverse total shoulder arthroplasty (RTSA) are necessary for complex and displaced fractures. Optimal treatment for PHFs remains complex and requires orthopedic surgeons to address numerous patient-specific factors [8].

Several systematic reviews have addressed medical treatments for PHFs [9, 10]. A meta-analysis conducted by Pizzo et al. identified 51 studies and found that RTSA was associated with higher Constant-Murley scores, improved active forward flexion, and a lower risk of complications than HA [9]. However, the study included both randomized controlled trials (RCTs) and observational studies, which might have caused overestimation of results. Another important meta-analysis performed by Davey et al. identified 13 RCTs and found that RTSA was associated with optimal functional outcomes and minimal revision rates [10]. However, two of the included trials examined the same population and recently published articles need to be included in a new meta-analysis to update the summary results. Network meta-analysis can synthesize data and obtain an estimate between the treatments of interest using indirect comparisons to rank various treatments for PHFs. Therefore, we performed a systematic review and network meta-analysis to update and expand previous systematic reviews, in order to inform clinical practice by comparing different types of surgical treatments for adult patients with PHFs.

## Materials and methods

### Search strategy and selection criteria

The PRISMA guidelines were used to guide the analysis and reporting of this systematic review and network meta-analysis [11]. Conservative therapy-controlled or head-to-head RCTs of four surgical techniques in patients with PHFs were eligible for inclusion, and publication language and status were not restricted. We systematically searched PubMed, Embase, and the Cochrane Library for eligible RCTs throughout June 2022, and the search terms mainly focused on “proximal humerus fractures” and “Randomized controlled trial”. Details of the search strategies are listed in the supplementary file. Trials from the ClinicalTrials.gov (US NIH) website that had already been completed, but the data not yet published, were also included. We also reviewed the reference lists of relevant original articles and reviews to identify eligible RCTs that met the inclusion criteria.

Two reviewers independently performed the literature search and study selection, and conflicts between reviewers were settled by discussion until a consensus was reached. The patients, intervention, control, outcomes, and study design (PICOS) criteria were applied to guide study selection: (1) patients: adult patients diagnosed with PHFs; (2) intervention: surgical treatments, including ORIF, IMN, HA, or RTSA; (3) control: nonsurgical treatment or any type of surgical treatment; (4) outcomes: Constant-Murley score (total score, pain, range of motion, strength, and activities of daily living), disabilities of the arm, shoulder and hand (DASH) score, European Quality of Life Five Dimensions (EQ-5D) score, secondary surgery, infection, avascular necrosis, nonunion, osteoarthritis, and other complications; and (5) study design: all eligible studies had to have an RCT design. Studies with the most informative and complete data were selected if the same study population was published more than once.

### Data collection and quality assessment

Two reviewers independently performed relevant information extraction and quality assessment of the included studies, and any disagreement was settled by an additional reviewer referring to the original article. The items collected from the included studies were as follows: first author’s surname, publication year, region, sample size, mean age, proportion of males, body mass index, dual-energy X-ray absorptiometry total body T-score, fracture pattern, intervention, control, follow-up duration, and reported outcomes. The methodological quality of the included RCTs was assessed using the Cochrane Collaboration risk of bias (random sequence generation, allocation concealment, blinding of participants and personnel, blinding of outcome assessment, incomplete outcome data, selective reporting, and other biases) [12].

### Statistical analysis

Continuous and dichotomous variables are presented as weighted mean differences (WMDs) and odds ratios (ORs), respectively. The heterogeneity of the network meta-analysis was assessed using the posterior distribution of the estimated heterogeneity variance and its predictive distribution [13]. Network meta-analysis was used to compare different medical treatments for PHFs in indirect and mixed comparisons [14]. Subsequently, the differences between direct and indirect estimates for a specific comparison in the loop were assessed using a loop-specific approach, and inconsistency was also assessed [15]. The assumption of consistency in the entire network was evaluated using the design-by-treatment interaction inconsistency model [14]. Given the heterogeneity among the included patients, the data analysis in our study was calculated using the inconsistent model, which considers the underlying variations across the included trials. The medical treatments for each outcome were compared and ranked using surface under the cumulative ranking (SUCRA) probabilities [16]. The WMD or OR with 95% credible intervals (CrIs) were applied to conduct pair-wise comparison analyses. Comparison-adjusted funnel plots were applied to assess publication bias, considering small-study effects [17]. All analyses were performed using the STATA software (version 14.0; Stata Corporation, College Station, TX, USA).

### Patient and public involvement

It was not appropriate or possible to involve patients or the public in the design, or conduct, or reporting, or dissemination plans of our research.

## Results

### Literature search and study selection

**Fig. 1:**
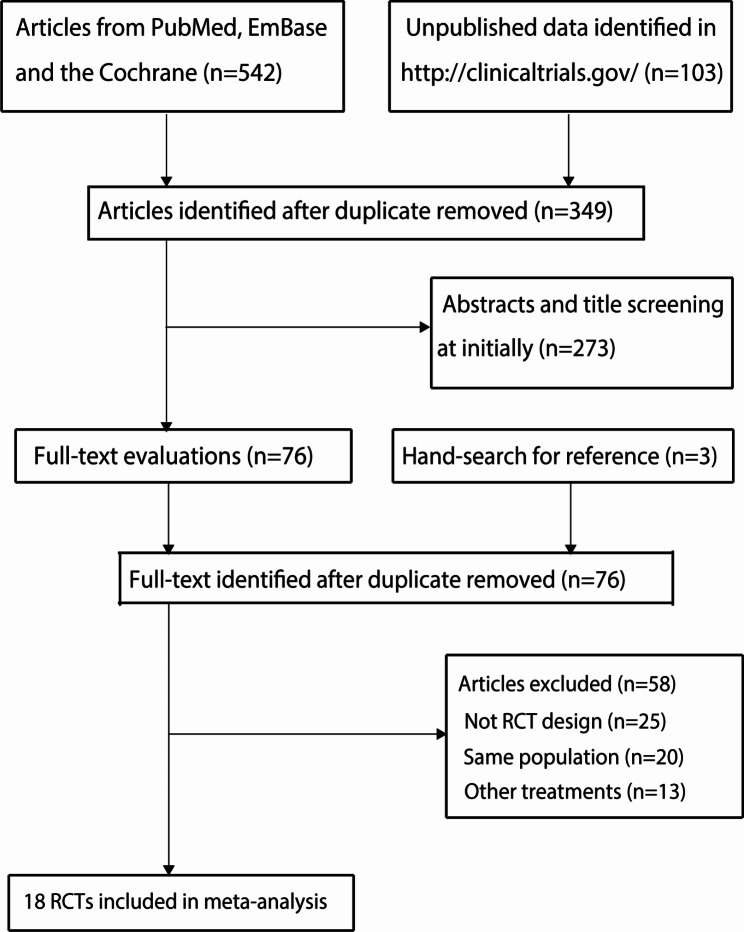
The PRISMA flowchart for the processes of literature search and study selection

A total of 542 articles were identified from the electronic databases, and 349 articles were retained after duplicate records were removed. An additional 273 studies were excluded because they reported irrelevant topics. The remaining 76 studies were retrieved for further full-text evaluation, and 58 studies were excluded. Reviewing the ClinicalTrials.gov (US NIH) website yielded 103 records, but none of these studies met the inclusion criteria. The reference lists of original articles were also reviewed and three trials for potential inclusion were identified, but all of these trials were already included from the database searches. Finally, 18 RCTs were selected for the final network meta-analysis [18–35], and the details of the study selection process are presented in Fig. 1.

### Study characteristics

The baseline characteristics of the included trials and involved patients are shown in Table 1. Of the 18 included studies, 1,182 patients with PHFs were involved, and the sample size ranged from 32 to 124. Fifteen studies were performed in Western countries [18–32] and the remaining three trials were performed in China [33–35]. The mean age of the included patients ranged from 49.0 to 83.5 years, while the proportion of males ranged from 6.0 to 53.7%. The methodological quality of the included studies is shown in Table S1. The overall quality of the included trials was low to moderate. Table S2 summarized the details of eligibility criteria and fracture type.

**Table 1:** The baseline characteristics of eligible trials and involved patients

Study	Region	Sample size	Age (years)	Male (%)	BMI (kg/m^2^)	DXA total body T score	Fracture pattern	Intervention	Control	Comparability of baseline characteristics	Follow-up (months)
Zyto 1997 [18]	Sweden	40 (20/20)	74.0	12.5	NA	NA	3, 4	ORIF (without strut graft)	Non-operative (sling for 7–10 days, then physiotherapy)	Balanced: age, gender, fracture, right: left side, dominant limb	50.0
Olerud 2011 [19]	Sweden	55 (27/28)	76.7	14.5	26.7	-1.5	4	HA	Non-operative (sling for 2 weeks, then rehabilitation regimen)	Balanced: age, gender, EQ-5D_index_ score, nondominant arm, BMI, DXA total body T score; imbalanced: cognitive function	24.0
Olerud 2011 [20]	Sweden	60 (30/30)	73.9	18.3	26.3	-1.3	3	ORIF (with strut graft)	Non-operative (sling for 2 weeks, then physiotherapy)	Balanced: age, gender, cognitive function, EQ-5D_index_ score, ADL, nondominant arm, BMI, DXA total body T score, and fracture type	24.0
Zhu 2011 [21]	China	51 (25/26)	52.6	33.3	NA	NA	2	IMN	ORIF (with strut graft)	Balanced: age, gender, dominant side, medial cortical comminution, interval between surgery and injury	36.0
Cai 2012 [22]	China	32 (13/19)	71.6	15.6	27.7	-1.2	4	ORIF (with strut graft)	HA	Balanced: age, gender, cognitive function, EQ-5D_index_ score, ADL, nondominant arm, BMI, DXA total body T score	24.0
Boons 2012 [23]	Netherlands	50 (25/25)	78.2	6.0	NA	NA	4	HA	Non-operative (sling for 6 weeks)	Balanced: age, gender, VAS pain, VAS disability	12.0
Fjalestad 2014 [24]	Norway	50 (25/25)	72.6	12.0	NA	NA	B2/C2	ORIF	Non-operative (sling for 2 weeks, then rehabilitation regimen)	Balanced: age, gender, injured arm, dominant arm, fracture types, pre-fracture medical conditions, concomitant injuries, pre-fracture 15 D score, Constant score	24.0
Sebastia-Forcada 2014 [25]	Spain	62 (31/31)	74.0	14.5	NA	NA	3, 4	RTSA	HA	Balanced: age, gender, dominant side, neer fracture, rotator cuff	28.5
Gracitelli 2016 [26]	Brazil	72 (36/36)	65.5	27.7	NA	NA	2, 3	IMN	ORIF (with strut graft)	Balanced: age, gender, smoking, dominant arm, mechanism of injury, interval between surgery and injury, intraoperative rotator cuff, neer classification, coronal deviation, medial cortical comminution	12.0
Chen 2016 [27]	China	60 (30/30)	66.0	46.7	NA	NA	4	ORIF (with strut graft)	HA	Balanced: age, gender, dominant side, dual mineral absorptiometry, accident, length of calcar segment, displacement of medial hinge, glenohumeral dislocation, varus impacted, valgus impacted, the mean time between trauma and surgery; imbalanced: medial comminution, blood loss	24.0
Launonen 2019 [28]	Finland, Sweden, Denmark, Estonia	88 (44/44)	72.5	9.1	NA	NA	2	ORIF (with strut graft)	Non-operative (sling for 3 weeks, then rehabilitation regimen)	Balanced: age, gender, fracture type, dominant side, smoking, diabetes, neurological diseases, DASH, Oxford Shoulder Score, EQ-5D_index_ score, 15D score	24.0
Lopiz 2019 [29]	Spain	59 (29/30)	83.5	13.6	NA	NA	3, 4	RTSA	Non-operative (sling for 3 weeks, then rehabilitation regimen)	Balanced: gender, dominant side, Charlson Comorbidity Index, fracture type; imbalanced: age	12.0
Plath 2019 [30]	Germany	68 (36/32)	75.6	25.0	NA	NA	3	IMN	ORIF (with strut graft)	Balanced: age, gender, right/left ratio, dominant side, ASA physical status classification, neer classification, AO classification	12.0
Fraser 2020 [31]	Norway	124 (64/60)	75.2	10.5	NA	NA	B2/C2	RTSA	ORIF (with strut graft)	Balanced: age, gender, living situation, diabetes, smoking, ASA class, time from injury to operation, OTA/AO fracture, injured arm, diminant arm; imbalanced: type of injury	24.0
Ramo 2020 [32]	Finland	82 (38/44)	49.0	53.7	27.9	NA	A/B/C	ORIF (with strut graft)	Non-operative (sling for 3 weeks, then rehabilitation regimen)	Balanced: age, gender, weight, height, BMI, smoking, radial nerve, AO/OTA classification, fracture location, injury mechanism, dominant limb, preinjury DASH score, optional work module, optional sports or performing arts module, preinjury 15D score	12.0
Helfen 2020 [33]	Germany	60 (30/30)	75.0	33.3	NA	NA	2	IMN	ORIF (with strut graft)	Balanced: age, gender, time to surgery	24.0
Jonsson 2021 [34]	Sweden	84 (41/43)	79.5	9.5	NA	NA	3, 4	RTSA	HA	Balanced: age, gender, right side dominant, dominant side injured, EQ-5D_index_ score, injury type, days from injury to surgery, duration of surgery in minutes, and study center	24.0
Boyer 2021 [35]	France	85 (43/42)	73.7	28.2	NA	NA	3, 4	IMN	ORIF (with strut graft)	Balanced: age, gender, injury type	66.0

*ADL: activities of daily living; BMI: body mass index; DASH: Disabilities of Arm, Shoulder, and Hand; DXA: dual-emission X-ray absorptiometry; HA: Hemi-Arthroplasty; IMN: Intramedullary nail; ORIF: Open reduction and internal fixation; RTSA: Reverse Total Shoulder Arthroplasty

The comparability of baseline characteristics are summarized in Table 1. Mostly factors between groups across included trials were balanced. However, Olerud et al. reported cognitive function between HA and conservative therapy was associated with statistically significant [19]. Chen et al. found significant differences between ORIF and HA for the medial comminution, and blood loss [27]. Lopiz et al. found the age between RTSA and conservative therapy was associated with statistically significant [29]. Fraser et al. found significant difference between RTSA and ORIF for the type of injury [31].

### Constant-murley score

**Fig. 2:**
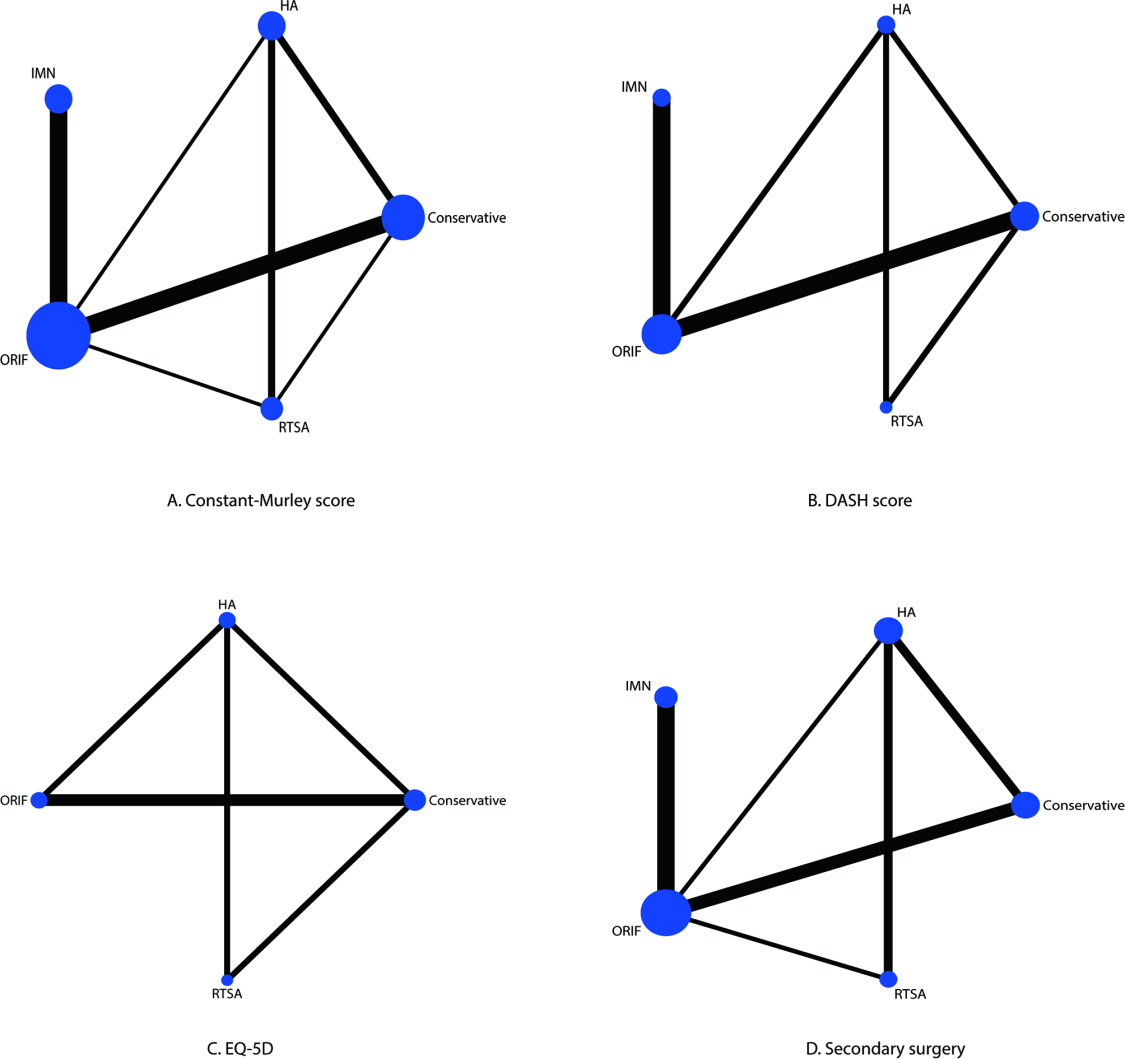
Network of comparisons for the Constant-Murley score **(A)**, DASH score **(B)**, EQ-5D **(C)**, and secondary surgery **(D)** included in the analysis. DASH: disabilities of the shoulder and hand; EQ-5D: European Quality of Life Five Dimensions

**Fig. 3:**
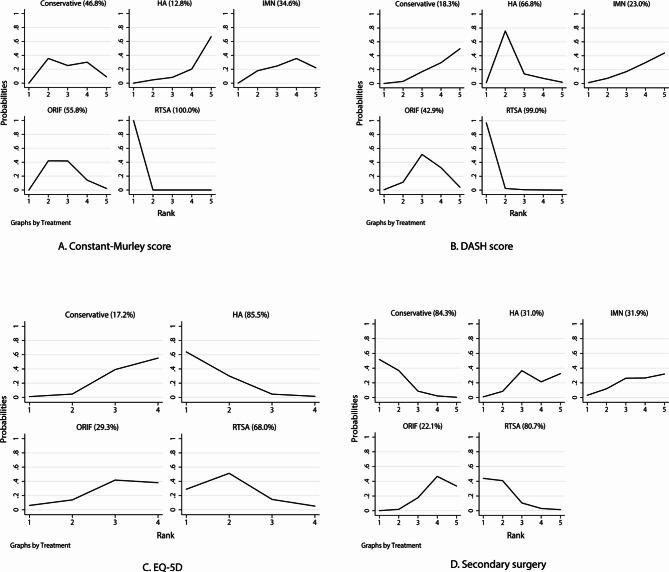
The surface under the cumulative ranking probabilities for the Constant-Murley score **(A)**, DASH score **(B)**, EQ-5D **(C)**, and secondary surgery **(D)**. DASH: disabilities of the shoulder and hand; EQ-5D: European Quality of Life Five Dimensions

**Fig. 4:**
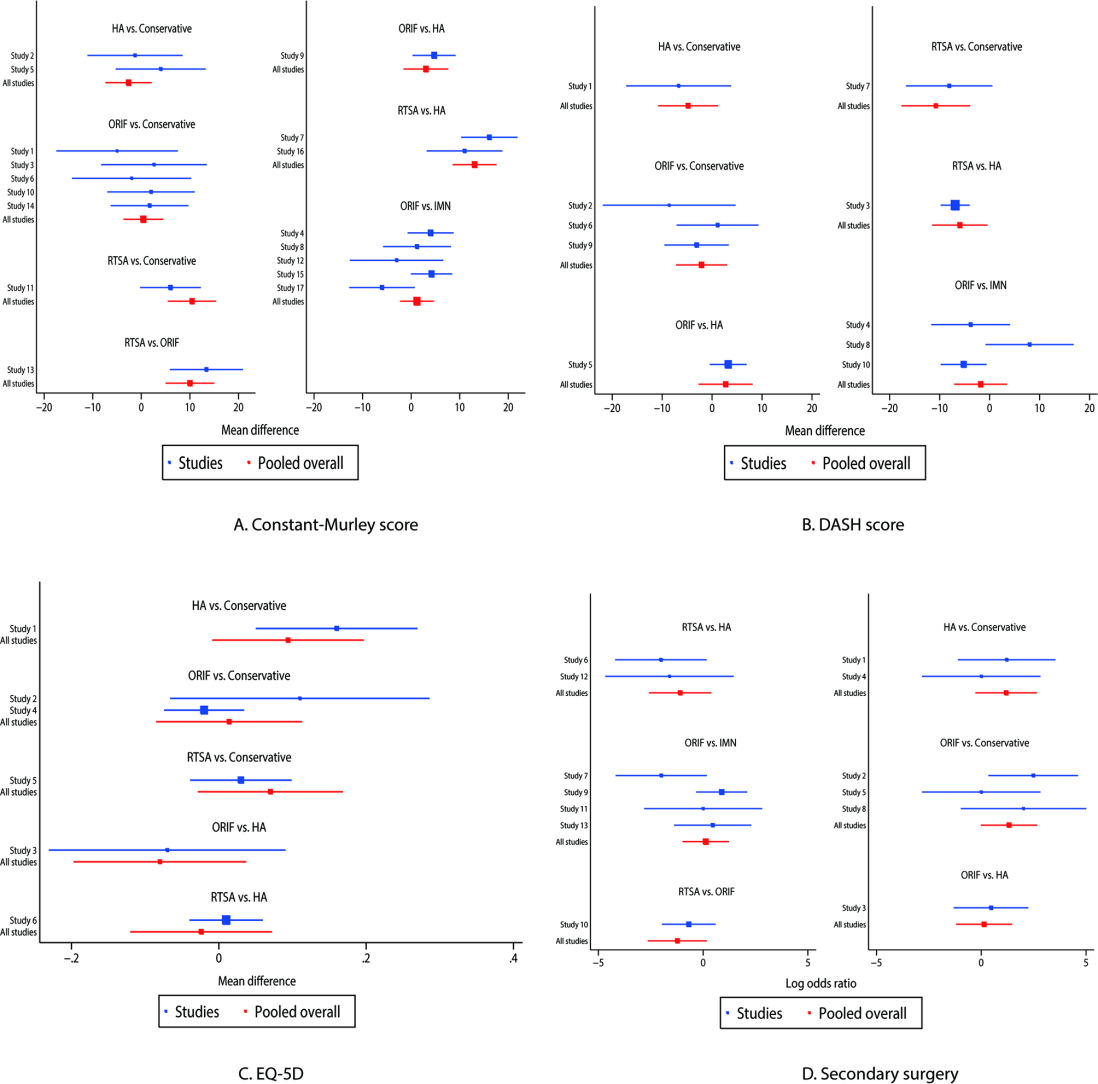
Pair-wise comparisons of treatments for the Constant-Murley score **(A)**, DASH score **(B)**, EQ-5D **(C)**, and secondary surgery **(D)**. DASH: disabilities of the shoulder and hand; EQ-5D European Quality of Life Five Dimensions

The network of eligible comparisons for the Constant-Murley score is shown in Fig. 2A. The number of trials for each comparison was weighted in nodes, and the precision of the direct estimate for each pairwise comparison was weighted in edges. The effects of medical treatments on the Constant-Murley score were compared and ranked using SUCRA probabilities. We noted that RTSA presented the best effect and highest Constant-Murley score (SUCRA: 100.0%; Fig. 3A). The pairwise comparisons results indicated that RTSA was associated with higher Constant-Murley scores than conservative therapy, ORIF, and HA (Fig. 4A). No significant publication bias for the Constant-Murley score was observed (Figure S1).

The network of eligible comparisons for pain scores, range of motion, strength score, and activities of daily living are shown in Figures S2-S5. The SUCRA probabilities indicated that RTSA presented the best effect on pain score (SUCRA: 92.4%; Figure S6), range of motion (SUCRA: 93.1%; Figure S7), strength score (SUCRA: 88.6%; Figure S8), and activities of daily living (SUCRA: 86.9%; Figure S9). Pairwise comparisons RTSA was associated with an elevated range of motion, or strength score, and improved activities of daily living as compared with HA (Figures S10-S13). No significant publication bias was observed in the pain score, range of motion, strength scores, and activities of daily living (Figures S14-S17).

### DASH score

The network of eligible comparisons for the DASH score is shown in Fig. 2B. The SUCRA probabilities indicated that RTSA (SUCRA: 99.0%) and HA (SUCRA: 66.8%) had relatively better effects on the DASH score (Fig. 3B). Figure 4B presents the results of pair-wise comparisons for the DASH score, which indicated that RTSA was associated with lower DASH scores than conservative therapy. There was no significant publication bias in the DASH scores (Figure S1).

### Health-related quality of life

The network of eligible comparisons for the EQ-5D is shown in Fig. 2C. We noted that the use of HA (SUCRA: 85.5%) and RTSA (SUCRA: 68.0%) had a relatively better effect on the EQ-5D (Fig. 3C). The pairwise comparison results for EQ-5D are shown in Fig. 4C, and there were no significant differences between the medical treatments regarding EQ-5D. No significant publication bias for EQ-5D was observed (Figure S1).

### Secondary surgery

The network of eligible comparisons for the risk of secondary surgery is shown in Fig. 2D. The SUCRA probabilities indicated that conservative therapy (SUCRA: 84.3%) and RTSA (SUCRA: 80.7%) had relatively better effects on the risk of secondary surgery (Fig. 3D). The pair-wise comparison results for the risk of secondary surgery are shown in Fig. 4D, and we noted that ORIF was associated with an increased risk of secondary surgery as compared with conservative therapy. No significant publication bias was observed in the risk of secondary surgery (Figure S1).

### Complications

The network of eligible comparisons for risk of infection, avascular necrosis, nonunion, and osteoarthritis are shown in Figures S18-S21. We noted that conservative therapy (SUCRA: 94.2%) and ORIF (SUCRA: 58.9%) had relatively better effects on the risk of infection (Figure S22). The use of HA (SUCRA: 78.1%) and IMN (SUCRA: 72.6%) had relatively better effects on the risk of avascular necrosis (Figure S23). The use of RTSA (SUCRA: 69.6%) and IMN (SUCRA: 66.2%) were associated with relatively lower risks of nonunion (Figure S24). The use of HA (SUCRA: 93.9%) had the best effect on the risk of osteoarthritis (Figure S25). The pair-wise comparison results indicated no significant differences between the medical treatments regarding the risk of infection, avascular necrosis, nonunion, and osteoarthritis (Figures S26-S29). No significant publication bias was observed in the risk of infection, avascular necrosis, nonunion, and osteoarthritis (Figures S30-S33).

### Other complications

Details of other complications are shown in Table S3. There were 426, 170, 175, 165, and 246 patients in the ORIF, IMN, HA, RTSA, and nonoperative groups, respectively. Of the 426 patients treated with ORIF, we found 25 screw penetration events, 11 loss of reduction events, eight impingement events, six refracture events, five stiffness events, four events of screws cut-through, three implant failure events, and three rotator cuff rupture events. Of the 170 patients treated with IMN, we noted eight loss of reduction events, six refracture events, six hardware problems, six events of malposition of implants, four rotator cuff rupture events, four stiffness events, three osteonecrosis events, and three events of tuberosity resorption/head migration. Of 175 patients treated with HA, we noted 10 events of secondary superior migration of the greater tuberosity, nine rotator cuff rupture events, six events of severe pain and limited function, five stiffness events, four refracture events, and four events of malpositioning of the greater tuberosity. Of the 165 patients treated with RTSA, we noted four nerve injury events, and four refracture events.

## Discussion

The current systematic review and network meta-analysis aimed to identify the optimal treatment for patients with PHFs. A total of 1,182 patients treated with ORIF, IMN, HA, RTSA, or conservative therapy from 18 RCTs were identified, and the characteristics of the patients varied broadly. Therefore, we applied an inconsistent model to the analysis of this study. We noted that RTSA had the best effect on the Constant-Murley score, including in the subscales of pain, range of motion, strength, and activities of daily living. Moreover, RTSA provided the best treatment effect on improvement in the DASH score. Furthermore, EQ-5D showed relatively better improvement in patients treated with HA and RTSA. In addition, the risk of secondary surgery was lower in patients treated with conservative therapy and RTSA. In terms of complications, conservative therapy and ORIF were associated with lower risks of infection, and infection in conservative therapy was rarely reported owing to it related to hematic spread form distant foci; HA and IMN were associated with lower risks of avascular necrosis; RTSA and IMN were associated with lower risks of nonunion; and the risk of osteoarthritis was lowest in patients treated with HA.

The current study found that RTSA offers the best effect on functional outcomes and secondary surgery, while health-related quality of life and risk of complications should be cautiously monitored. RTSA is widely used to manage complex PHFs, especially for patients with 3-part and 4-part PHF. In this study, mostly included studies involved patients with 3-part and 4-part PHF, which could explained the best effect on functional outcomes for RTSA. Studies have already demonstrated the use of RTSA as a salvage procedure in patients for whom treatment with HA, ORIF, or conservative therapy fails [36–39]. Moreover, RTSA had a relatively better health-related quality of life, however, RTSA produced lower scores on the EQ-5D than did HA. Furthermore, RTSA was associated with higher risks of infection and avascular necrosis. Moreover, the incidences of nerve injury (risk: 2.42%) and refracture (risk: 2.42%) should be cautiously monitored. The risk of nerve injury related to RTSA can be explained by brachial plexus stretch injuries that occur during the positioning of the arm at the extremes of motion [40, 41]. In addition, limited preoperative external rotation followed by extreme external rotation during humeral and glenoid preparation are associated with an increased risk of nerve injury [42]. However, pair-wise comparisons did not find significant differences between groups, which could be explained by the lower incidence of complications, and which should be further verified in large-scale real-world studies.

The use of ORIF had a moderate effect on the Constant-Murley score, while it was associated with poor DASH and EQ-5D scores, and secondary surgery. Moreover, ORIF was associated with higher risks of avascular necrosis and nonunion. However, ORIF treatment of PHFs in the elderly resulted in an arm that was generally durable once healed. Studies have already demonstrated that the use of ORIF is associated with an increased risk of secondary surgery, with a prevalence ranging from 13 to 29% [43, 44]. The main causes of secondary surgery include hardware failure, screw cutout, nonunion, malunion, infection, and avascular necrosis [43, 45]. Moreover, nearly 16% of ORIF patients reported experiencing long-term shoulder symptoms [45]. Finally, we noted that the risks of screw penetration (5.87%), loss of reduction (2.58%), impingement (1.88%), and refracture (1.41%) were relatively higher, which could explain the high risk of secondary surgery for patients treated with ORIF. The risk of screw penetration was higher for patients treated with locked-plate technology, and the prevalence ranged from 0 to 43%, especially in patients aged older than 60 years who had a 3- or 4-part fracture [46]. The narrow diameter of the metacarpal bone and the need for early motion are associated with an increased risk of loss of reduction [47]. Additionally, improper product installation can affect the risks of screw penetration, loss of reduction, and impingement. Finally, screw penetration, impingement, failure of prosthesis fixation or implantation, and malreduced fracture could be affected by the design of the device, and the mechanical properties of device could affect the failure of the prosthesis after implantation, such as via prosthesis release, loosening, displacement, fracture re-displacement, and loss of reduction.

Our study found that IMN was associated with a poor effect on functional outcomes, while it had a moderate effect on the risk of secondary surgery. Moreover, IMN appears to associate with a lower risk of complications, except in the case of osteoarthritis. The potential reason for this could be that IMN is widely used in 2- or 3-part fractures [48], whereas most patients involved in this study had 3- or 4-part fractures. Furthermore, the use of IMN was associated with less invasion and better preservation of soft tissue envelopes, which ensures blood supply to the bony fragments with acceptable bony alignment. These results could explain the relatively lower risk of complications in IMN. However, the inherent drawbacks of IMN include increased comminution, inadequate compression and stability, shoulder pain and stiffness, disorder of the rotator cuff, and back-out of proximal screws [49], which could explain the poor functional outcomes of IMN for PHFs. Finally, the risks of loss of reduction (4.71%), refracture (3.53%), hardware problems (3.53%), and malposition of implants (3.53%) were relatively high, which may increase the risk of secondary surgery. The length of the screw plays an important role in the risks of backing out, loss of reduction, and perforation of the glenoid [50].

The use of HA was associated with a poor Constant-Murley score, while it provided relatively better effects on the DASH and EQ-5D scores. Moreover, the number of complications in patients treated with HA was lower, except in the case of infection. A previous study suggested that HA should be applied to patients at high risk of complications, especially osteonecrosis and implant failure [51]. Moreover, the use of HA has been found to associate with moderate postoperative joint function, and the rate of nonunion of the large tubercles was higher [52, 53]. Finally, the risks of secondary superior migration of the greater tuberosity (5.71%) and rotator cuff rupture (5.14%) were high, and the risk of greater tuberosity migration could be caused by the design of the humeral prosthesis, which causing rise number of patients did not selected HA [53, 54]. Thus, early preventive strategies should be applied in patients treated with HA.

Several limitations of this study should be acknowledged. First, most of the included trials had low to moderate quality, and the results might be biased by uncontrolled confounders. Second, the severity of PHFs varied across the included trials, which could affect the medical treatments and prognoses of patients. Third, background therapies and postoperative rehabilitation were not addressed, which could also have affected prognosis. Fourth, the surgical and conservative treatments are differing across included trials owing to various disease status, which could affect the net effect estimates of ORIF, IMN, HA, and RTSA. Finally, there are inherent limitations to the meta-analysis of published articles, including inevitable publication bias and restricted detailed analyses.

## Conclusion

This study found that RTSA offers relatively better effects on joint functional outcomes and secondary surgery, while the risk of complications should be cautiously monitored. The optimal treatments for PHFs were assessed in our study according to each investigated outcome, and appropriate treatments for PHFs should consider patient-specific factors.

### Electronic supplementary material

Below is the link to the electronic supplementary material.

Supplementary Material 1

Supplementary Material 2

## Data Availability

The datasets used and/or analysed during the current study are available from the corresponding author on reasonable request.

## Abbreviations

PHFs: Proximal humerus fractures
ORIF: open reduction internal fixation
IMN: intramedullary nailing
HA: hemiarthroplasty
RTSA: reverse total shoulder arthroplasty
PICOS: patients, intervention, control, outcomes, and study design
DASH: disabilities of the arm, shoulder and hand
EQ-5D: European Quality of Life Five Dimensions
WMDs: weighted mean differences
ORs: odds ratios
SUCRA: surface under the cumulative ranking
CrIs: credible intervals
