# Supplementary material for: Comparative efficacy and safety of medical treatments for proximal humerus fractures: a systematic review and network meta-analysis
Source: BMC Musculoskelet Disord. 2024 Jan 2;25:17. doi: 10.1186/s12891-023-07053-x (PMC10759392; doi:10.1186/s12891-023-07053-x)
Supplement: Supplementary file 2 — Supplementary Material 2 [file 12891_2023_7053_MOESM2_ESM.docx]

1. **Search strategy**

**PubMed:**

#1. “Humeral Fractures”[Mesh]

#2. “proximal humeral”[All Fields]

#3. “humeral fracture”[All Fields]

#4. OR/#1-3

#5. “randomized controlled trial”[Publication Type]

#6. “randomized”[Title/Abstract]

#7. “controlled”[Title/Abstract]

#8. “trial”[Title/Abstract])

#9. AND/#6-8

#10. OR/#5, #9

#11. AND/#4 and #10

**Cochrane Library**

#1. MeSH descriptor Shoulder Fractures explode all trees

#2. MeSH descriptor Humeral Fractures explode all trees

#3. MeSH descriptor Humerus explode all trees

#4.(shoulder* OR humer*)

#5. fract*

#6. (#3 OR #4)

#7. (#1 OR #2)

#8. (#5 AND #6)

#9. (#7 OR #8)

**EMBASE**

#1. Humerus Fracture/

#2. ((humer$ or shoulder$) adj10 (fract$ or fixat$)).tw

#3. OR/#1-2

#4. (proximal or neck$1 or sub?capital).tw

#5. AND/#3-4

#6. exp Randomized Controlled Trial/

#7.exp Double Blind Procedure/

#8.exp Single Blind Procedure/

#9.exp Crossover Procedure/

#10.Controlled Study/

#11. OR/#6-10

#12. ((clinical or controlled or comparative or placebo or prospective$ or randomi#ed) adj3 (trial or study)).tw.

#13.(random$ adj7 (allocat$ or allot$ or assign$ or basis$ or divid$ or order$)).tw

#14.((singl$ or doubl$ or trebl$ or tripl$) adj7 (blind$ or mask$)).tw.

#15.(cross?over$ or (cross adj1 over$)).tw

#16.((allocat$ or allot$ or assign$ or divid$) adj3 (condition$ or experiment$ or intervention$ or treatment$ or therap$ or control$ or group$)).tw

#17. OR/#12-16

#18. or/#11, 17

#19. limit #18 to human

#20. AND/#5,19

Table S1. Quality assessment of included trials

| Study | Random sequence generation | Allocation concealment | Blinding of participants and personnel | Blinding of outcome assessment | Incomplete outcome data | Selective reporting | Other bias |
| --- | --- | --- | --- | --- | --- | --- | --- |
| Zyto 1997 [18] | Low | Low | High | High | High | High | Low |
| Olerud 2011 [19] | Low | Low | High | High | High | High | Low |
| Olerud 2011 [20] | Low | Low | High | High | Low | High | Low |
| Zhu 2011 [21] | Low | High | High | High | Low | High | Low |
| Cai 2012 [22] | Low | High | High | High | High | High | Low |
| Boons 2012 [23] | Low | Low | High | High | Low | High | Low |
| Fjalestad 2014 [24] | Low | Low | High | High | Low | High | Low |
| Sebastia-Forcada 2014 [25] | Low | Low | High | Low | Low | High | Low |
| Gracitelli 2016 [26] | Low | High | High | High | High | High | Low |
| Chen 2016 [27] | Low | Low | High | High | Low | High | Low |
| Launonen 2019 [28] | Low | Low | High | Low | Low | High | Low |
| Lopiz 2019 [29] | Low | High | High | High | Low | High | Low |
| Plath 2019 [30] | Low | Low | High | High | Low | High | Low |
| Fraser 2020 [31] | Low | Low | High | Low | Low | High | Low |
| Ramo 2020 [32] | Low | Low | High | Low | Low | High | Low |
| Helfen 2020 [33] | Low | High | High | High | High | High | Low |
| Jonsson 2021 [34] | Low | Low | High | Low | Low | High | Low |
| Boyer 2021 [35] | Low | High | High | High | Low | High | Low |

Table S2. The summary results for the type of fracture and eligibility criteria

| Study | Eligibility criteria | Type of fracture |
| --- | --- | --- |
| Zyto 1997 [18] | A displaced three- or four-part fracture of the humerus not caused by high-energy trauma and not pathological; at least 30% contact between the humeral head and the humeral shaft; no other fractures elsewhere in the upper limbs; no concomitant disease likely to influence the end result; and ability of the patient to co-operate. | Fracture: three-part: 19/18; four-part: 1/2; Right:left: 6/14; 8/12 |
| Olerud 2011 [19] | Age 55 years or older, acute displaced 4-part fracture of the surgical neck of the humerus, a fracture sustained after a low-energy trauma, no previous shoulder problems, independent living conditions, and no severe cognitive  dysfunction | Four-part: 100% |
| Olerud 2011 [20] | Age 55 years or older, acute displaced 3-part fracture of the surgical neck of the humerus, a fracture sustained after a low-energy trauma, no previous shoulder problems, independent living conditions, and no severe cognitive  dysfunction | Three-part: 100% |
| Zhu 2011 [21] | Skeletally mature patient who agreed to participate in the study, a twopart surgical neck fracture of the proximal part of the humerus as classified with the Neer system, and an acute fracture treated surgically within twenty-one days after the injury | Two-part: 100% |
| Cai 2012 [22] | Age 67 years or older, acute displaced 4-part fracture of the surgical neck of the humerus, a fracture sustained after a lowenergy trauma, no previous shoulder problems, independent living conditions, and no severe cognitive dysfunction | Four-part: 100% |
| Boons 2012 [23] | 65 years or older who had displaced proximal humeral fourpart fractures | Four-part: 100% |
| Fjalestad 2014 [24] | Aged 60 or above with displaced proximal humeral fracture (AO/OTA group B2 or C2) | Fracture types: B2/C2, 13/12; 13/12. Right:left: 13/12; 15/10 |
| Sebastia-Forcada 2014 [25] | Aged 70 years or older with an acute proximal humeral fracture who were candidates  for shoulder arthroplasty | Fracture: three-part: 5/4; four-part: 26/26 |
| Gracitelli 2016 [26] | Age between 50 and 85 years and a PHF with displacement ≥1 cm or ≥45° of angulation between the head and diaphysis of the humerus, with or without involvement of the greater tuberosity, and treated surgically ≤21 days after the injury | Fracture: two-part: 16/16; three-part: 16/17 |
| Chen 2016 [27] | Bone mineral density was less than –3.0, and suffered from an acute fourpart PHF, and/or fracture dislocation were in accordance with the inclusive criteria | Four-part: 100% |
| Launonen 2019 [28] | Patients aged 60 years or over with displaced 2-part low-energy PHF occurring less than 2 weeks before allocation and treatment onset | Two-part: 100%, surgical neck |
| Lopiz 2019 [29] | Aged 80 years or older with a 3- or 4-part displaced PHF available for follow-up for at least 12 months. The patients had to be able to understand the informedconsent process of the study. | Fracture: three-part: 4/5; four-part: 25/25 |
| Plath 2019 [30] | Age > 60 years, diagnosed with PHF and the capacity to give informed consent | Fracture: two-part: 5/4;three-part: 25/24; four-part: 6/4 |
| Fraser 2020 [31] | Patients who were 65 to 85 years of age and presented with a severely displaced proximal humeral fracture of type B2 or C2 | Fracture types: B2/C2, 26/29; 38/31. Right:left: 35/29; 32/28 |
| Ramo 2020 [32] | Age: 18 years or older, unilateral displaced humeral shaft fracture, displacement was at least the amount of the thickness of the cortex or in transverse fractures diastasis  of the half of the thickness of the cortex was required, the fracture was lying in a zone delimited proximally by the superior border of the pectoralis major tendon attachment and distally by the line lying 5 cm from the upper border of the olecranon fossa as evaluated from the x-ray, the fracture was less than 10 days old, the patient was willing to accept both treatment options and willing to participate in all follow-up visits, patient spoke and read fluently either Finnish or Swedish | Fracture types: A/B/C: 34/4/0; 36/7/1 |
| Helfen 2020 [33] | Age: ≥60 years, or female postmenopausal, 2-part fracture according to AO-classification: AO 11-A3, signed informed consent, patient can read and understand German | Two-part: 100% |
| Jonsson 2021 [34] | A displaced 3- or 4-part proximal humeral fracture, age over 70 years, independent living and a low-energy injury mechanism | Fracture: three-part: 20/20; four-part: 19/16 |
| Boyer 2021 [35] | Patients with a 3- and 4-part displaced acute proximal humeral fractures | Fracture: three-part: 36/29; four-part: 7/13 |

Table S3. The summary results for adverse events

| Study | Intervention | Control |
| --- | --- | --- |
| Zyto 1997 [18] | ORIF: infection: 2; K-wire penetration: 1; pulmonary embolus: 1; AVN: 1; nonunion: 1; osteoarthritis: 4 | Non-operative: osteoarthritis: 2 |
| Olerud 2011 [19] | HA: A mean secondary dislocation of the greater tubercle: 5; complete resorption of the greater tubercle: 1; partial resorptions: 2; 3 additional surgery (secondary screw penetration: 1; impingement and Stiffness: 1; redisplacement of the greater Tubercle: 1) | Non-operative: additional surgery (1 patient presented complete displacement of the shaft without bony contact was detected and treated with HA); nonunion: 1; AVN: 3; osteoarthritis: 5 |
| Olerud 2011 [20] | ORIF: AVN: 3; infection: 2; nonunion: 1; impingement: 1; stiffness: 3; secondary screw penetration: 3 | Non-operative: impingement: 1; nonunion: 1; AVN: 2; osteoarthritis: 1 |
| Zhu 2011 [21] | ORIF: heterotopic ossification: 2; screw penetration: 5; pneumothorax: 1 | IMN: heterotopic ossification: 1 |
| Cai 2012 [22] | ORIF: nonunion: 1; fixation failure: 2 | HA: dislocation: 1; infection: 1; prosthesis loosening: 1 |
| Boons 2012 [23] | HA: head-stem separation: 1; malpositioning of the greater tuberosity: 4; secondary superior migration of the greater tuberosity: 5 (partial resorption of bone: 2); proximal migration with an acromiohumeral distance less than 7 mm: 1; nonunion of the greater tuberosity: 2 | Non-operative: osteonecrosis of the head with consequent radiographic narrowing; nonunion: 3; pain and impairment of the affected shoulder: 1 |
| Fjalestad 2014 [24] | ORIF: sinking of the humeral head with subsequently penetration of the screw tips into the joint space: 7; post-traumatic glenohumeral osteoarthrosis: 1; pull-out of the plate: 1; death: 2 | Non-operative: re-displacement of the fracture: 1; nonunion: 2 |
| Sebastia-Forcada 2014 [25] | RTSA: hematoma: 1; deep wound infection:1 | HA: severe pain and limited function: 6; An acromion-head distance of ≤7 mm, reflecting insufficiency or rupture of the rotator cuff: 8; intraoperative humeral fracture: 1; superficial infection: 1; postoperative stiffness: 1 |
| Gracitelli 2016 [26] | IMN: complex regional pain syndrome: 1; infection: 1; loss of reduction of greater tuberosity: 1; loss of reduction of humeral head: 3; hardware problems: 6; osteonecrosis: 2; complete rotator cuff tears: 4; shoulder stiffness: 4 | ORIF: insufficient reduction:1; loss of reduction of humeral head: 1; hardware problems: 1; refracture: 1; complete rotator cuff tears: 2; shoulder stiffness: 2 |
| Chen 2016 [27] | ORIF: loss reduction (varus displacement):1; AVN: 1; screw perforation: 1 | HA: superficial infection: 2; shoulder stiffness: 4; tuberosity migration: 2 |
| Launonen 2019 [28] | ORIF: peri-implant fracture: 1; implant failures: 2 | Non-operative: none |
| Lopiz 2019 [29] | RTSA: suprascapular nerve injury: 2 | Non-operative: none |
| Plath 2019 [30] | IMN: malposition of implants: 4; loss of reduction of humeral head with screw cut-out: 2; loss of reduction of humeral head w/o screw cut-out: 2; tuberosity resorption/head migration: 3; migration without tuberosity resorption: 1; osteonecrosis of humeral head: 1; secondary surgery: 5 | ORIF: malposition of implants: 2; loss of reduction of humeral head with screw cut-out: 8; loss of reduction of greater tuberosity: 1; tuberosity resorption/head migration: 2; osteonecrosis of humeral head: 1; axillary nerve lesion: 1; adhesive capsulitis: 1; secondary surgery: 7 |
| Fraser 2020 [31] | RTSA: nerve injury: 2; deep wound infection: 2; periprosthetic fracture or fracture distal to plate: 2; perioperative glenoid fracture: 1 | ORIF: screw penetration: 9; periprosthetic fracture or fracture distal to plate: 1; nonunion: 1; rotator cuff rupture: 1 |
| Ramo 2020 [32] | ORIF: cardiac arrhythmia: 1; secondary temporaty radial nerve palsy: 3; superficial wound infection: 2; wound seroma: 1; shoulder adhesive capsulitis: 1 | Non-operative: pulmonary embolism: 1; fracture nonunion: 11; refracture: 1; secondary temporaty radial nerve palsy: 1; superficial wound infection: 1; shoulder adhesive capsulitis: 1; loss of reduction: 1; sensory disturbance in the forearm: 1 |
| Helfen 2020 [33] | IMN: malreduced fracture: 5; implant malpositioning: 2; periimplant fracture: 1 | ORIF: malreduced fracture: 4; screw-cut out: 2 |
| Jonsson 2021 [34] | RTSA: death: 1; distal humeral fracture: 1; complex regional pain syndrome: 1 | HA: periprosthetic humeral fracture: 3; proximal migration of the humeral head: 1 |
| Boyer 2021 [35] | IMN: nonunion: 2; screws cut-through: 2; AVN: 2; humeral impingement: 2; infection: 1 | ORIF: nonunion: 4; screws cut-through: 4; AVN: 6; humeral impingement: 7; infection: 1 |

**
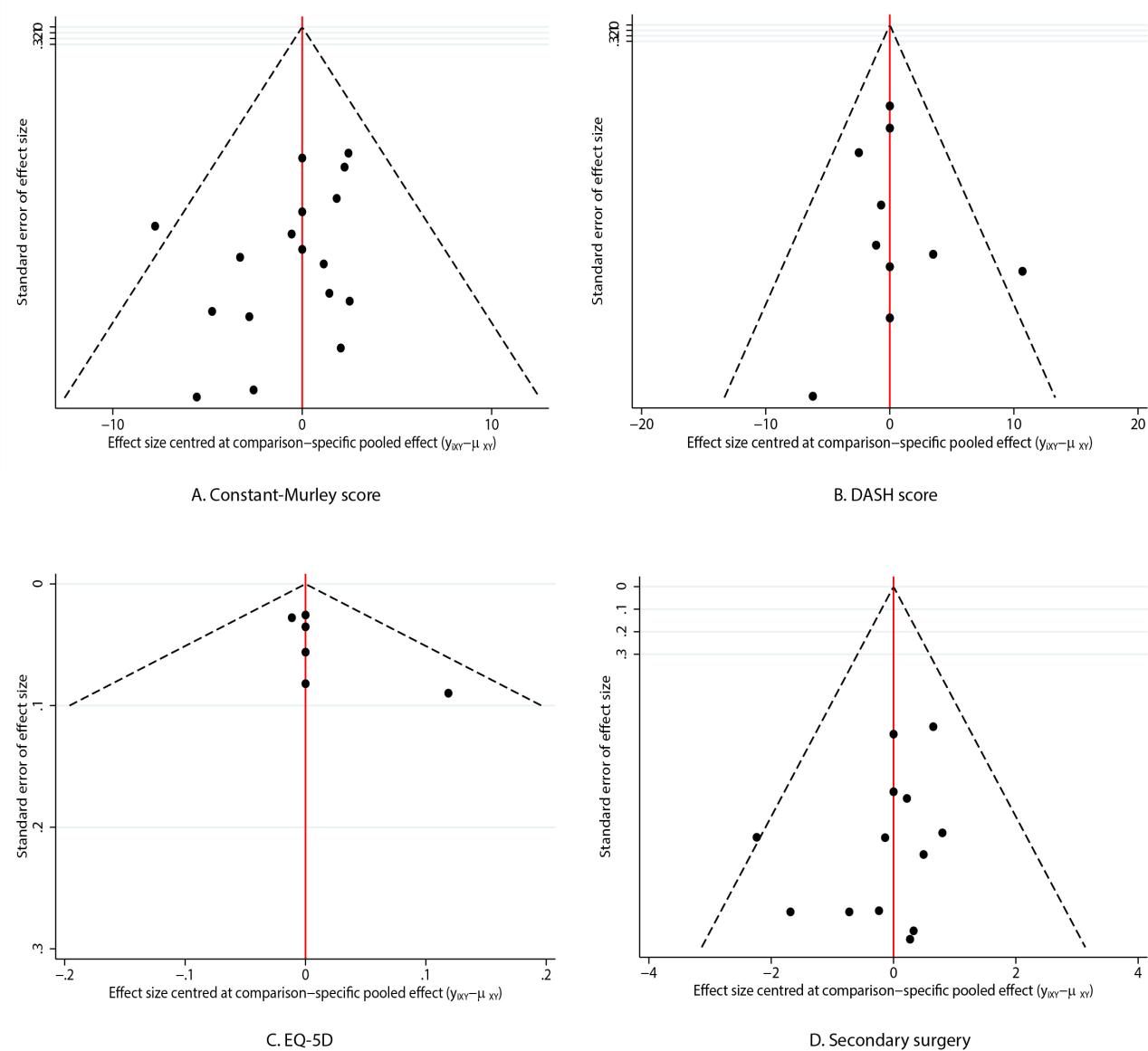
**

Figure S1. Funnel plot for the Constant-Murley score (A), DASH score (B), EQ-5D (C), and secondary surgery (D).

DASH, disabilities of the shoulder and hand; EQ-5D, European Quality of Life Five Dimensions


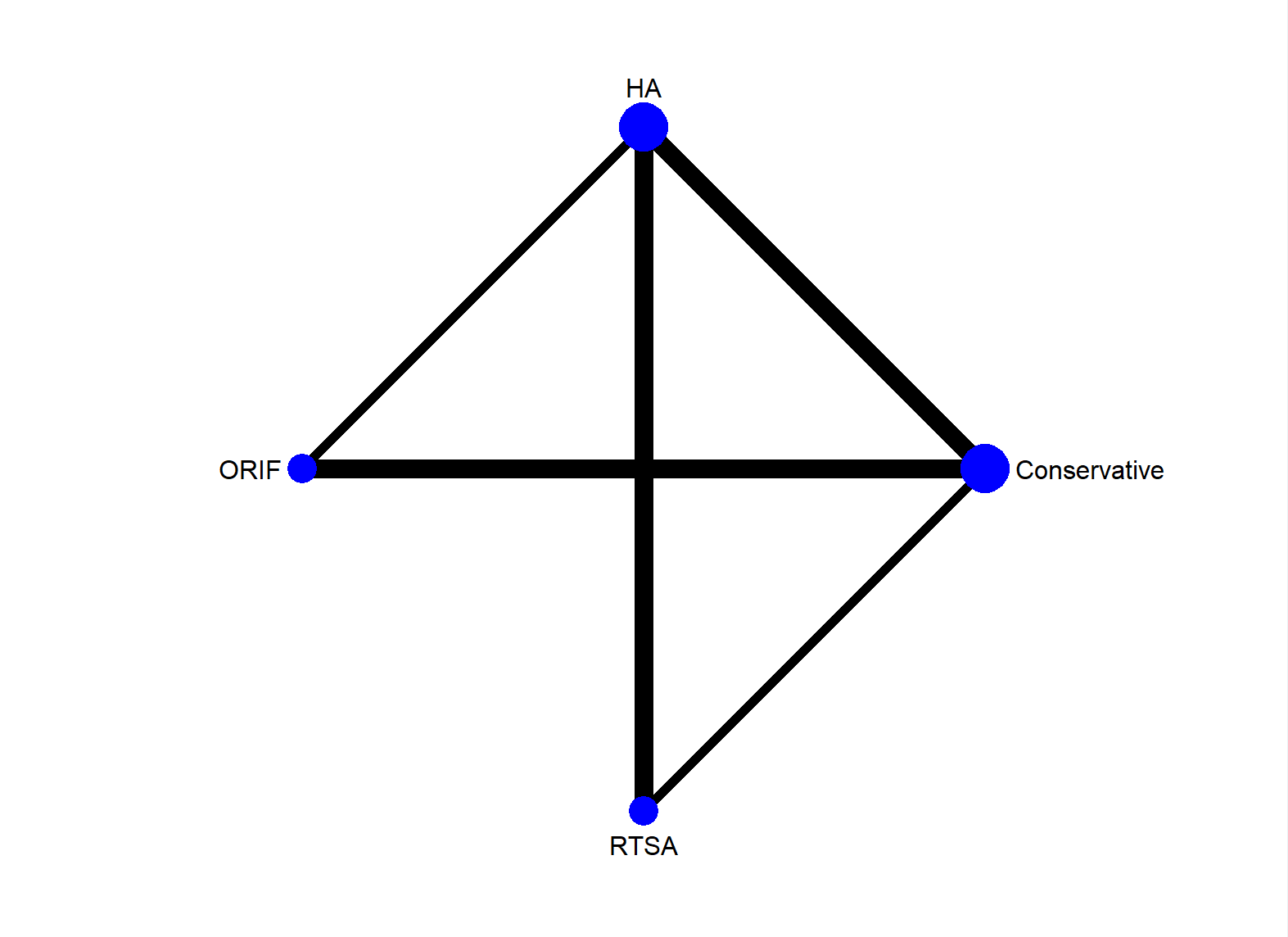


Figure S2. Network of comparisons for pain included in the analysis.


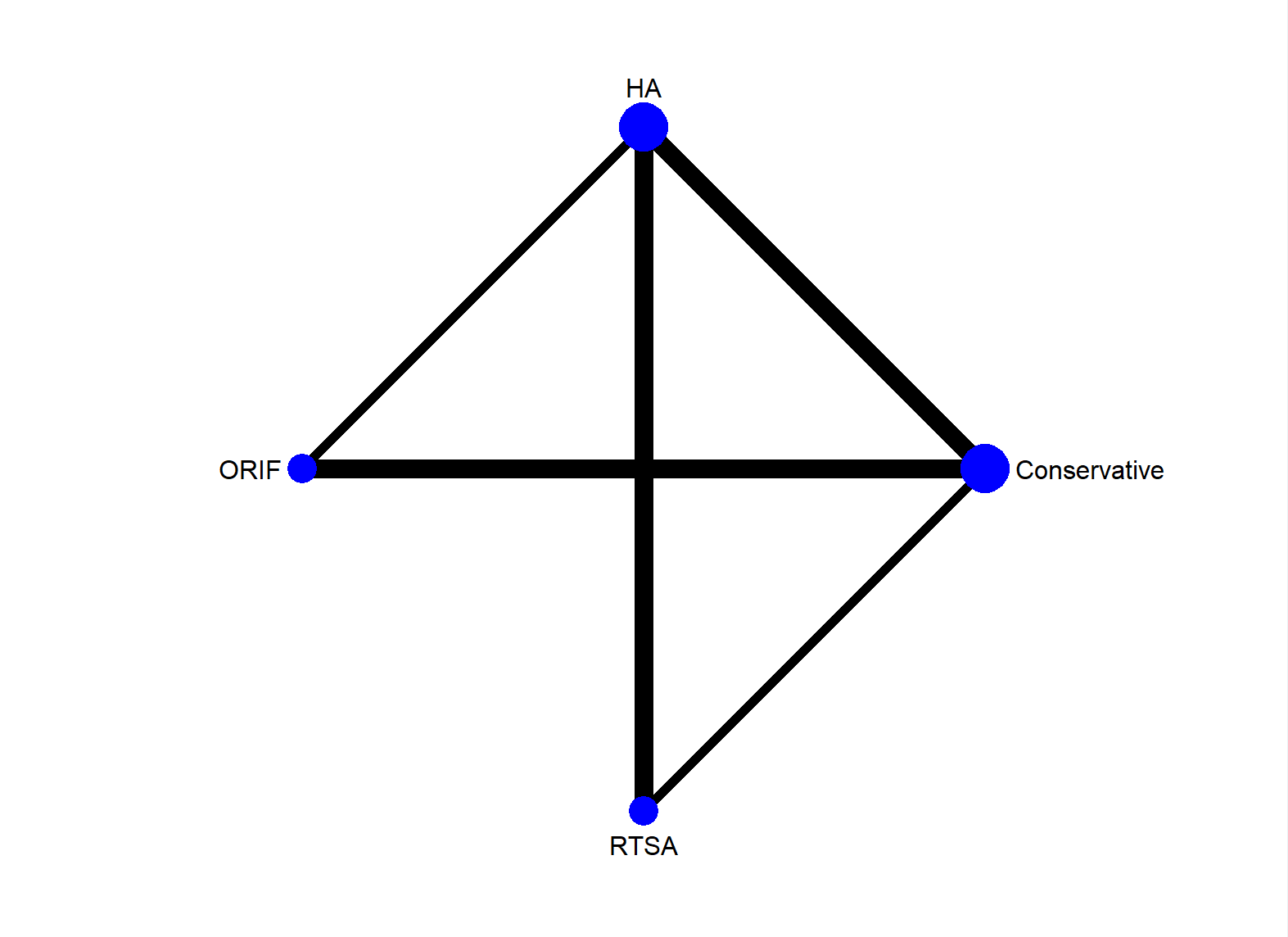


Figure S3. Network of comparisons for range of motion included in the analysis.


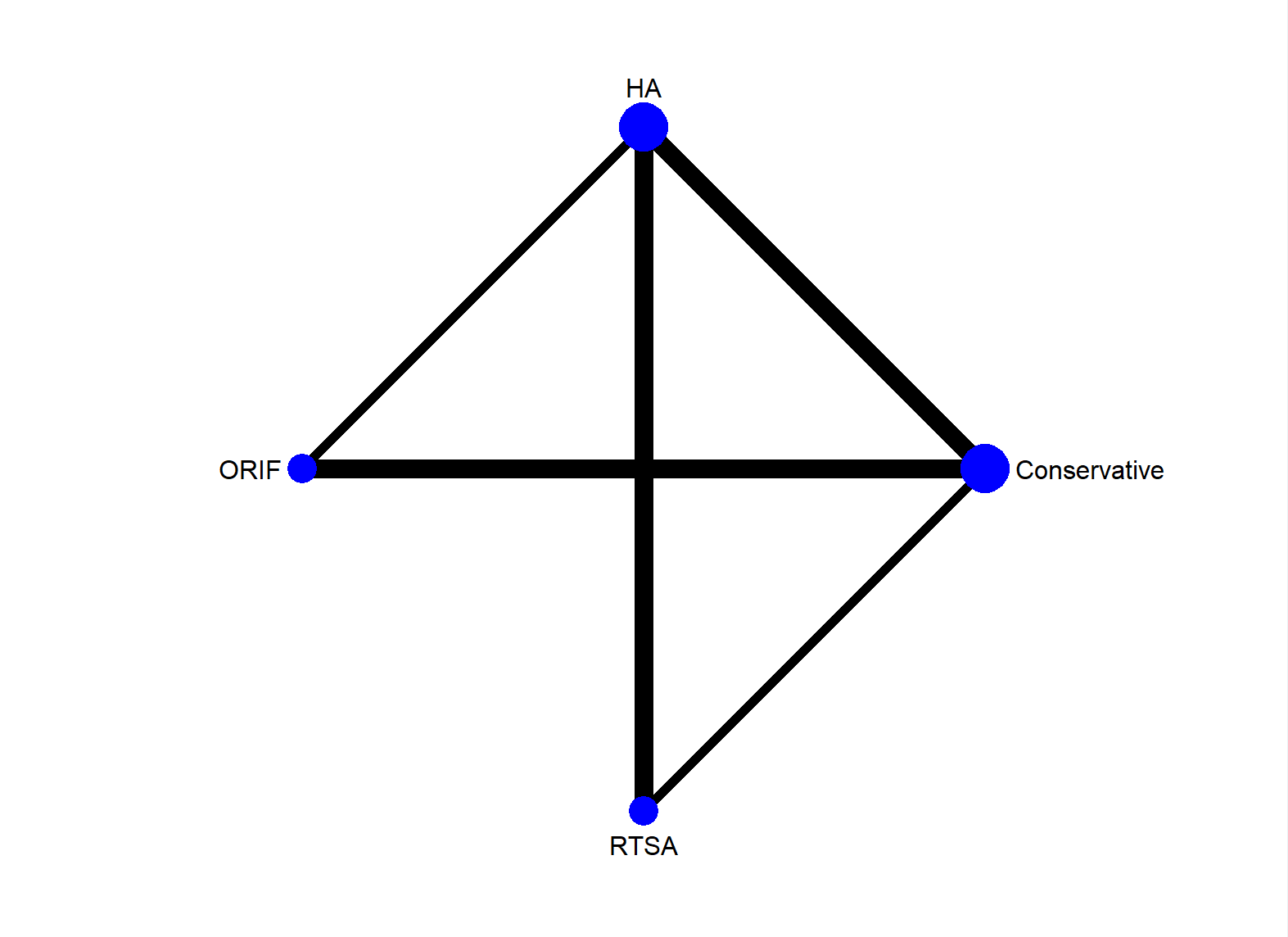


Figure S4. Network of comparisons for strength included in the analysis.


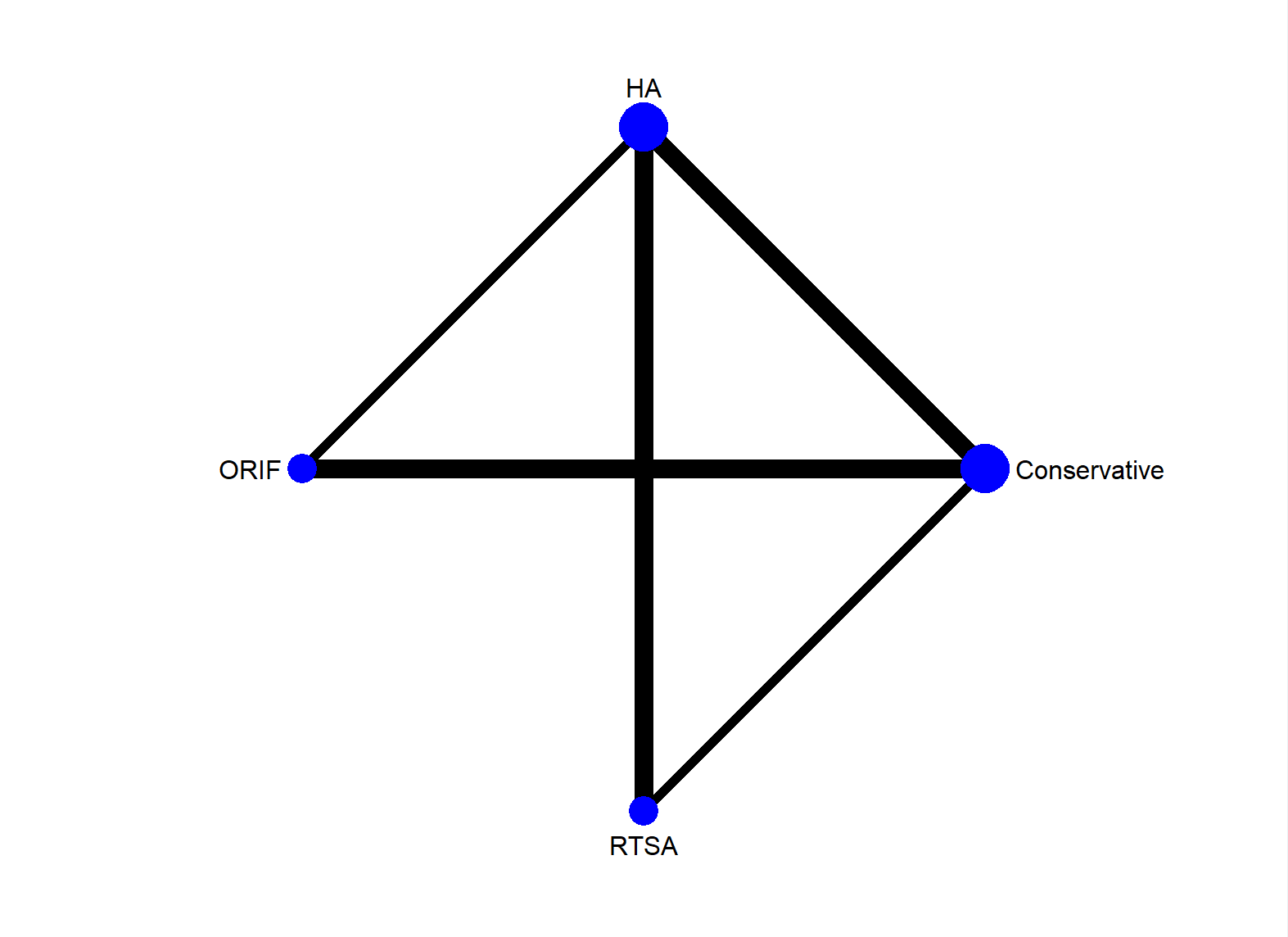


Figure S5. Network of comparisons for activity of daily living included in the analysis.


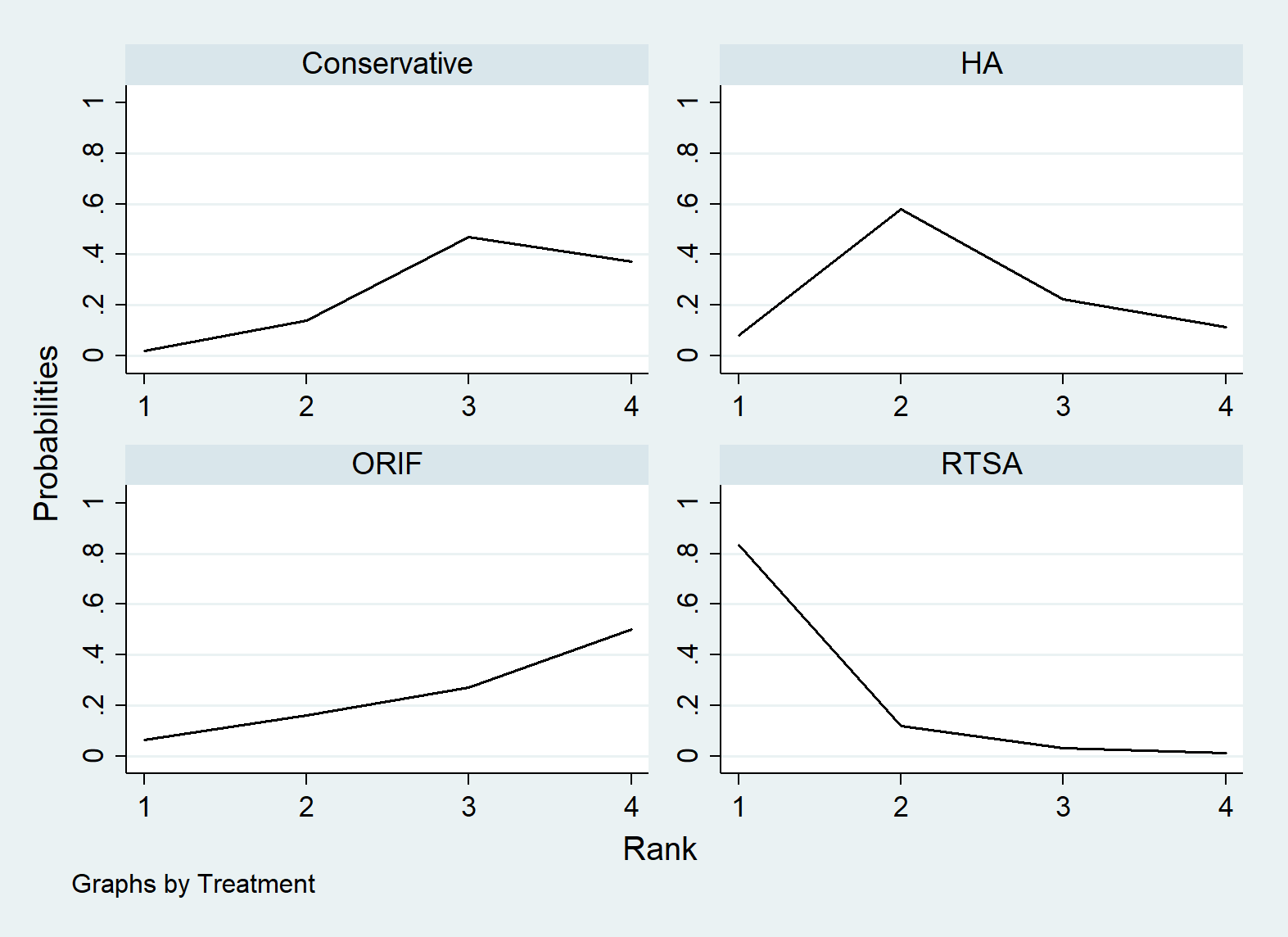


Figure S6. The SUCRA rank test for pain. Conservative: 26.9%; HA: 54.2%; ORIF: 26.5%; RTSA: 92.4%


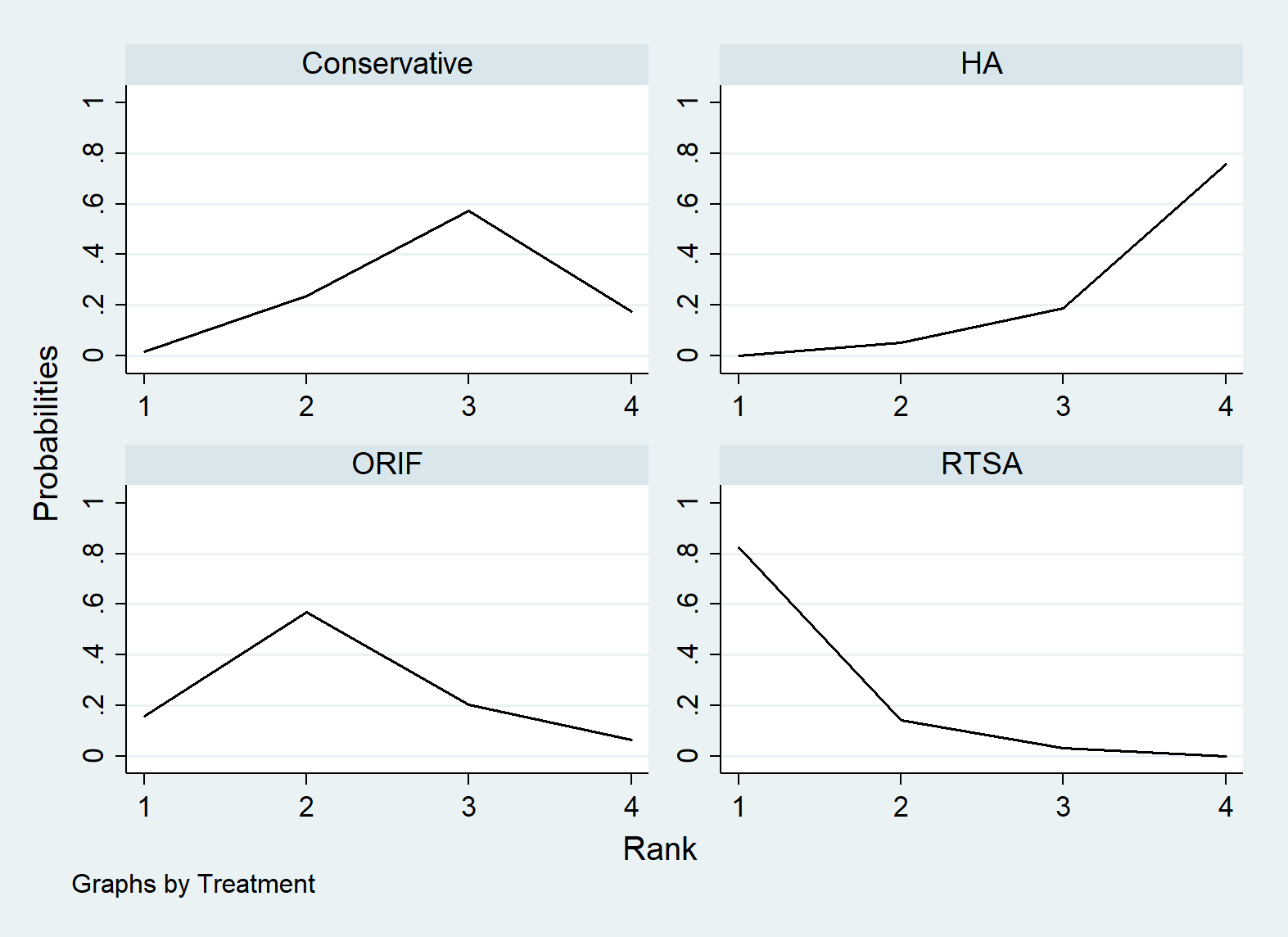


Figure S7. The SUCRA rank test for range of motion. Conservative: 36.4%; HA: 9.8%; ORIF: 60.8%; RTSA: 93.1%


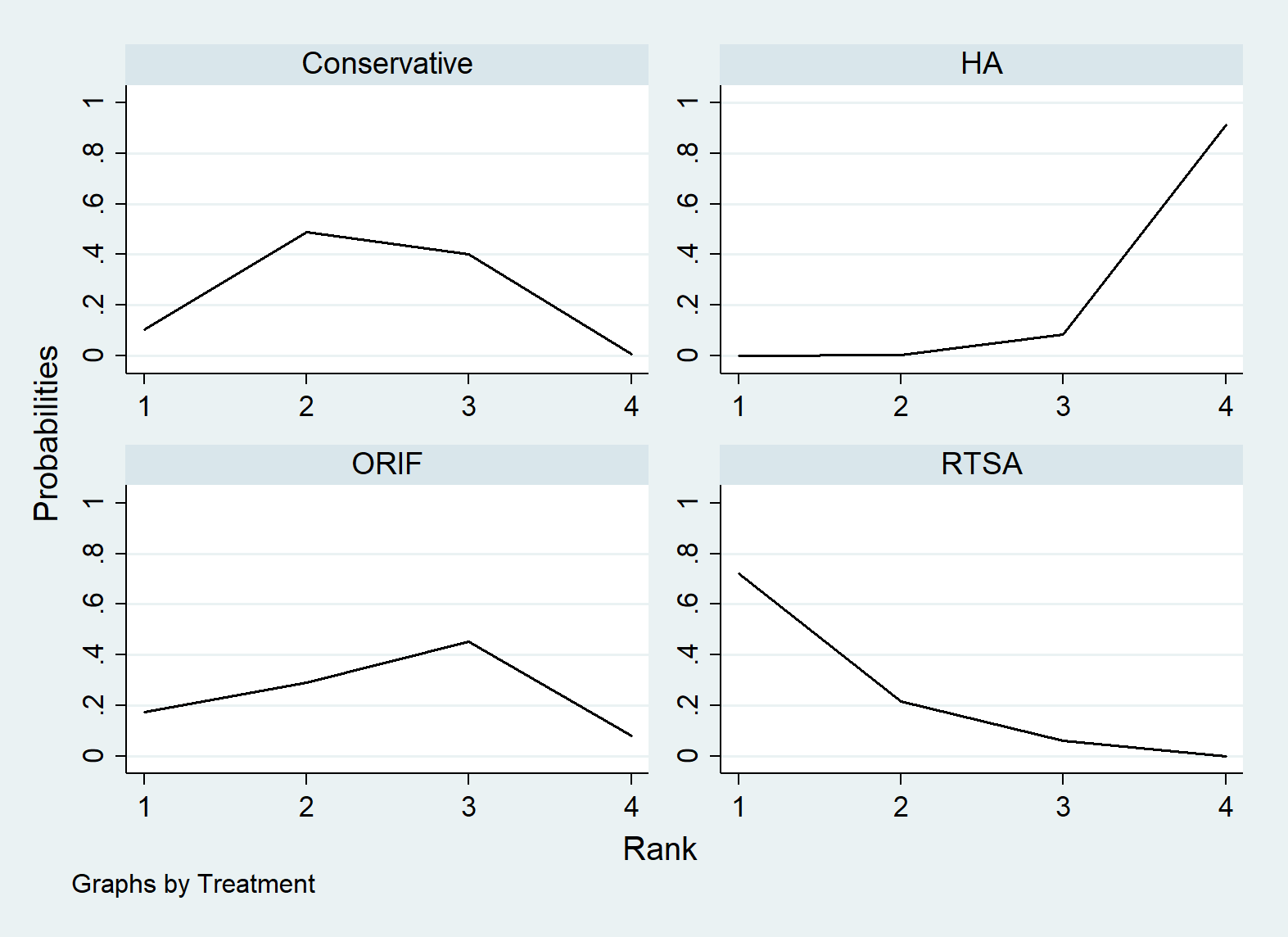


Figure S8. The SUCRA rank test for strength. Conservative: 56.2%; HA: 3.0%; ORIF: 52.1%; RTSA: 88.6%


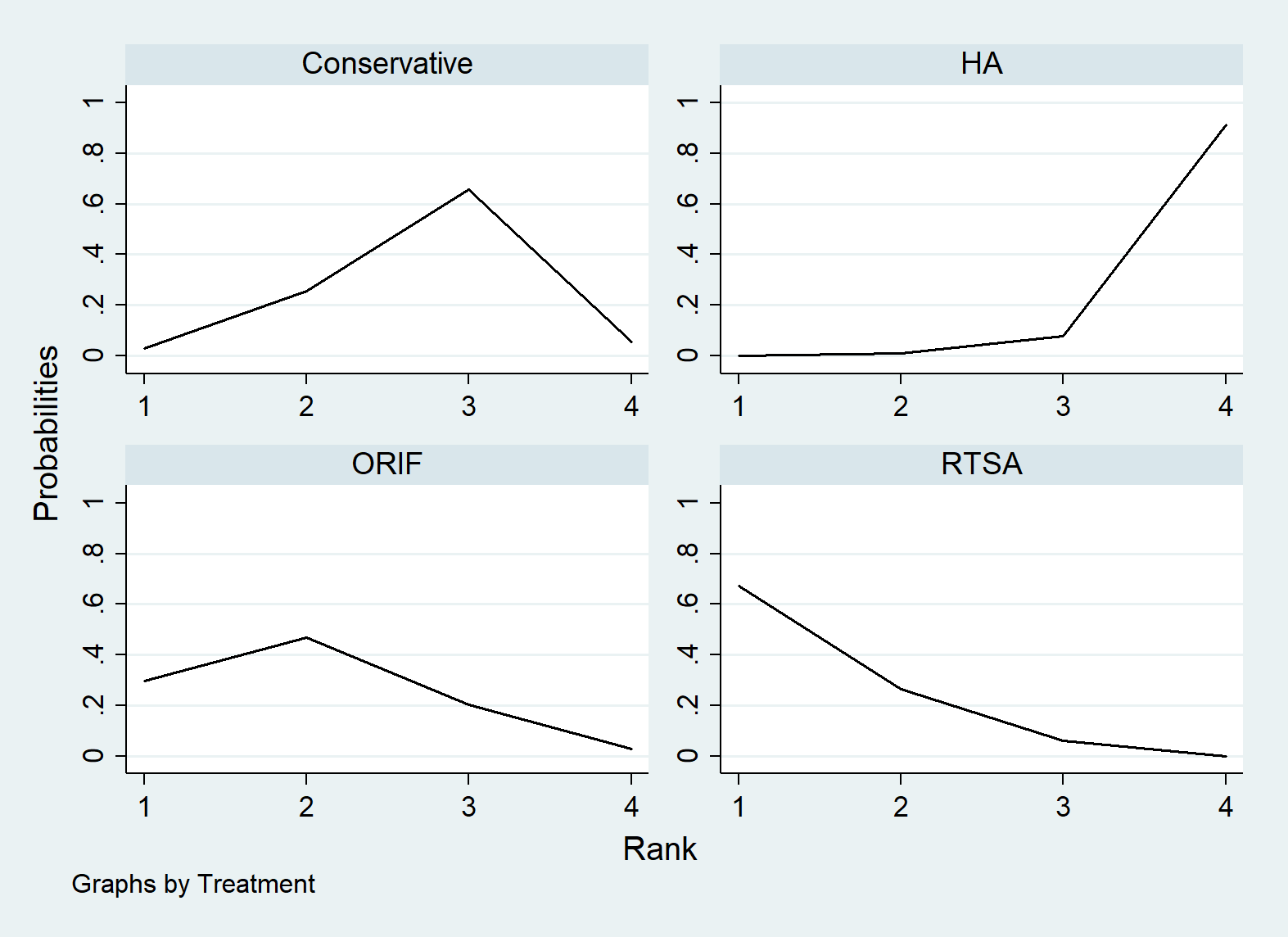


Figure S9. The SUCRA rank test for activity of daily living. Conservative: 41.9%; HA: 3.3%; ORIF: 67.8%; RTSA: 86.9%


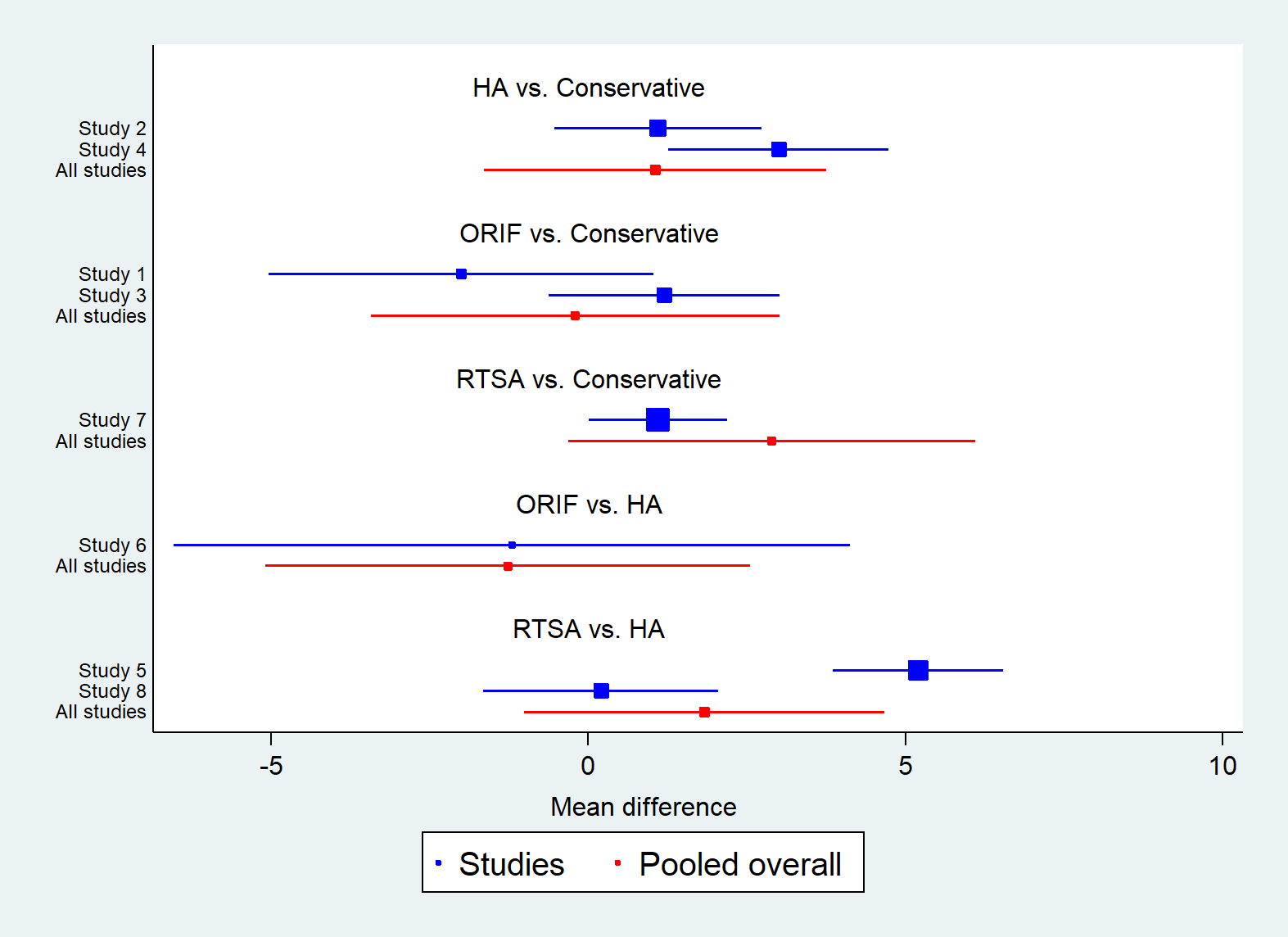


Figure S10. The pair-wise comparisons agents for pain


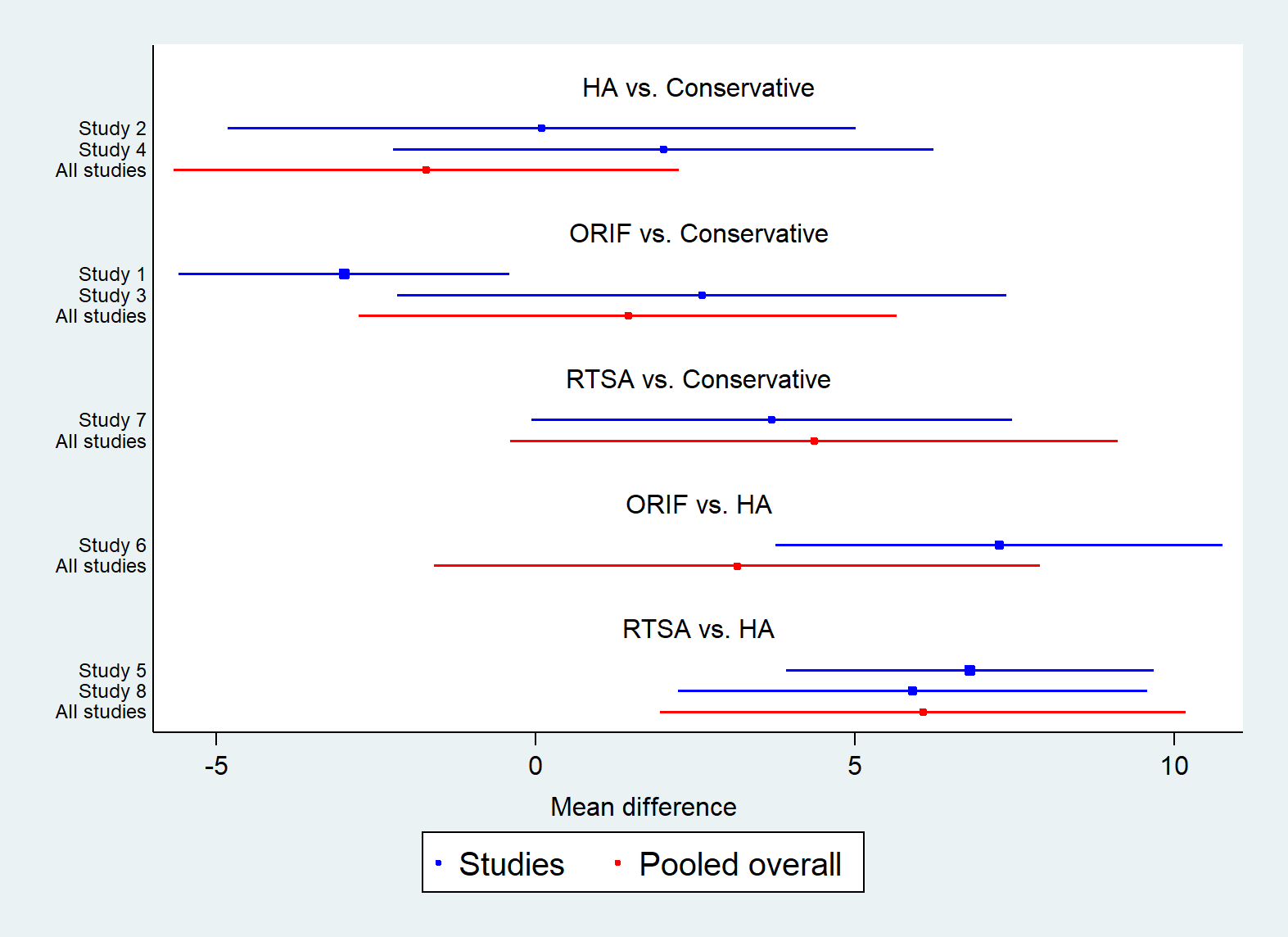


Figure S11. The pair-wise comparisons agents for range of motion.


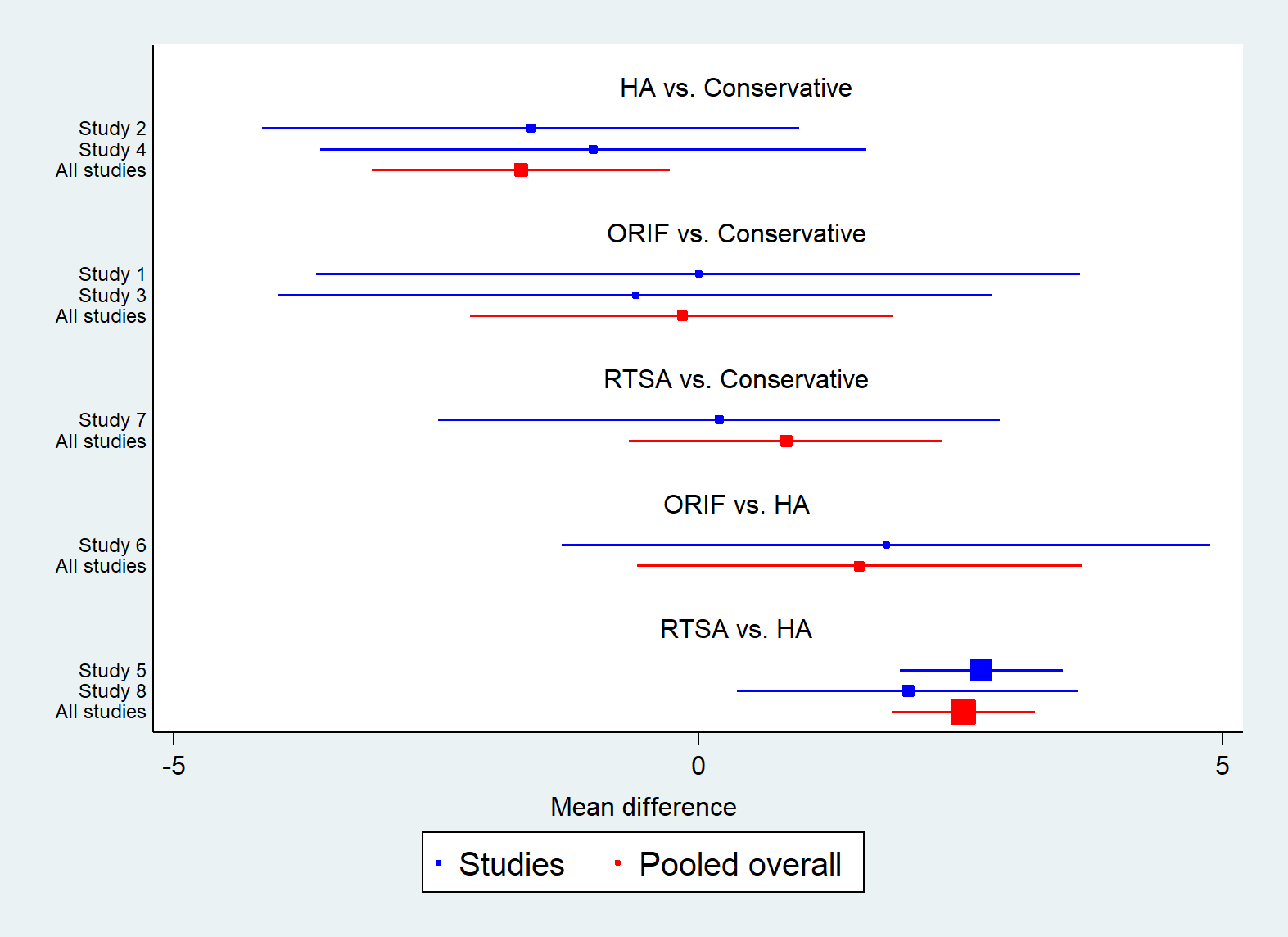


Figure S12. The pair-wise comparisons agents for strength.


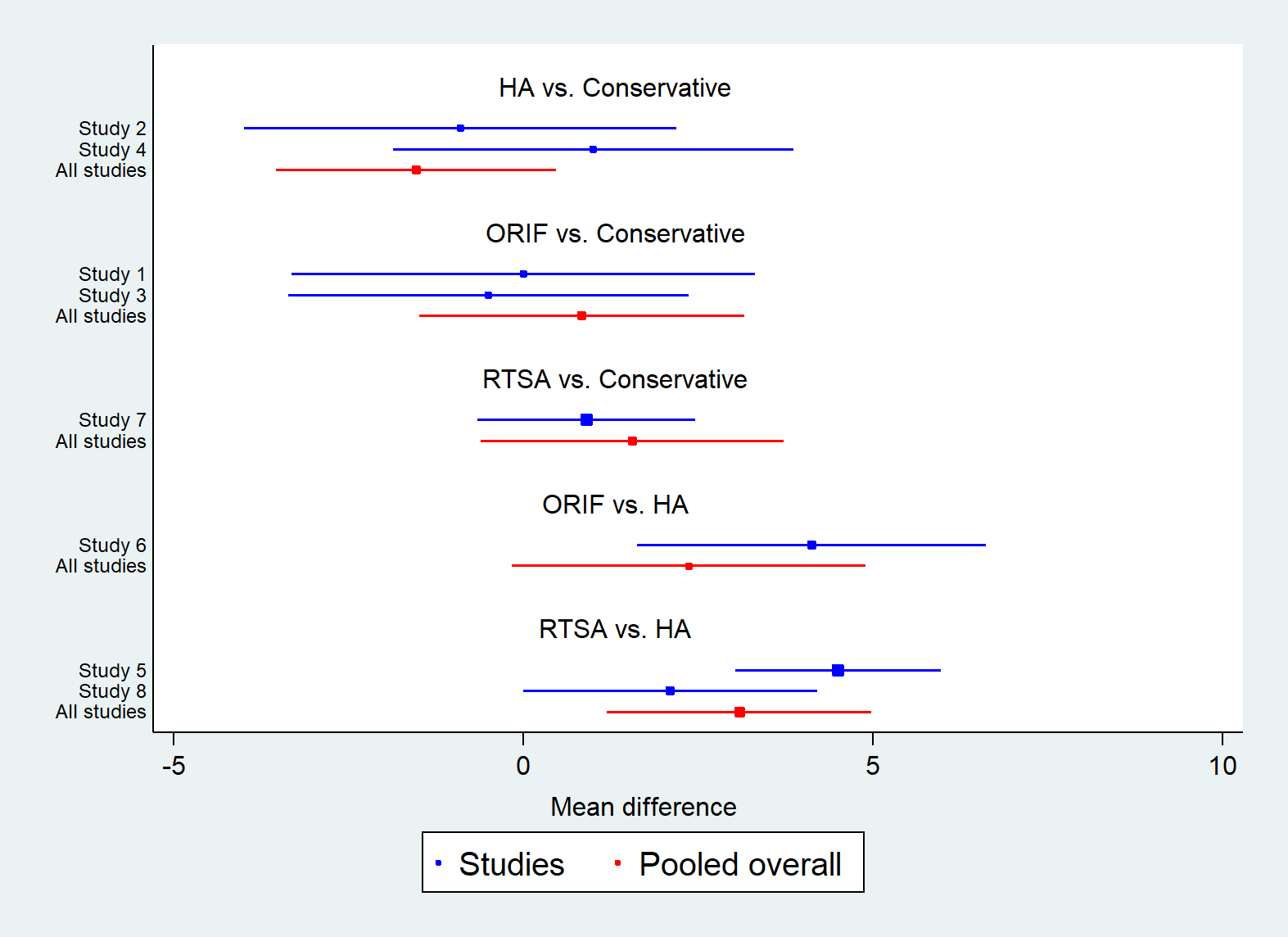


Figure S13. The pair-wise comparisons agents for activity of daily living.


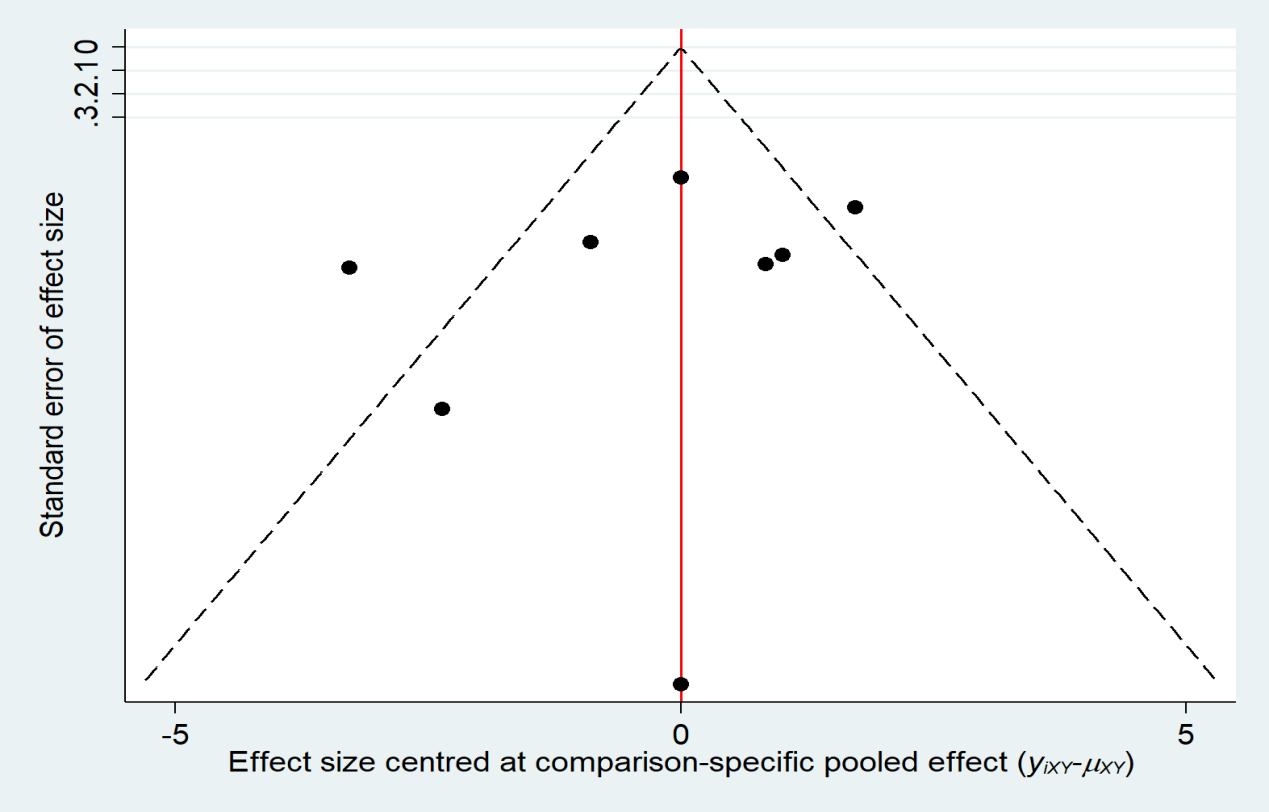


Figure S14. Funnel plot for pain


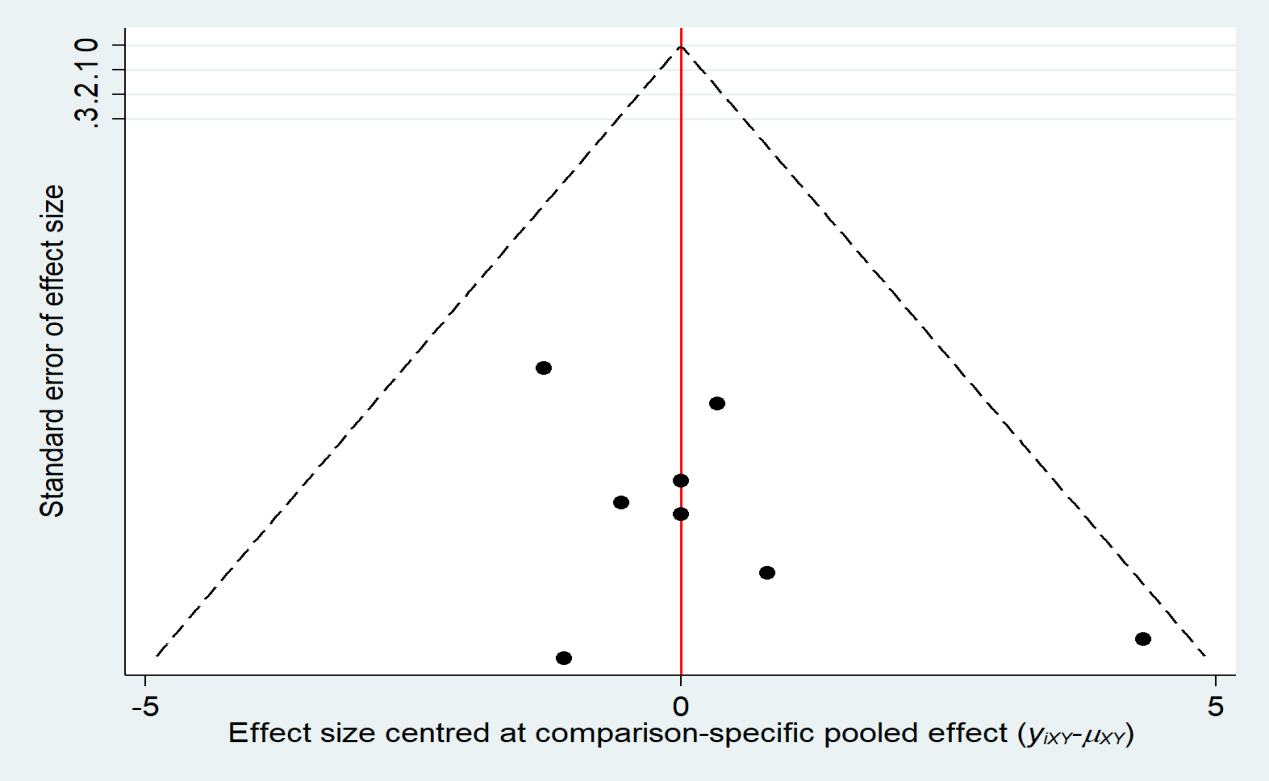


Figure S15. Funnel plot for range of motion.


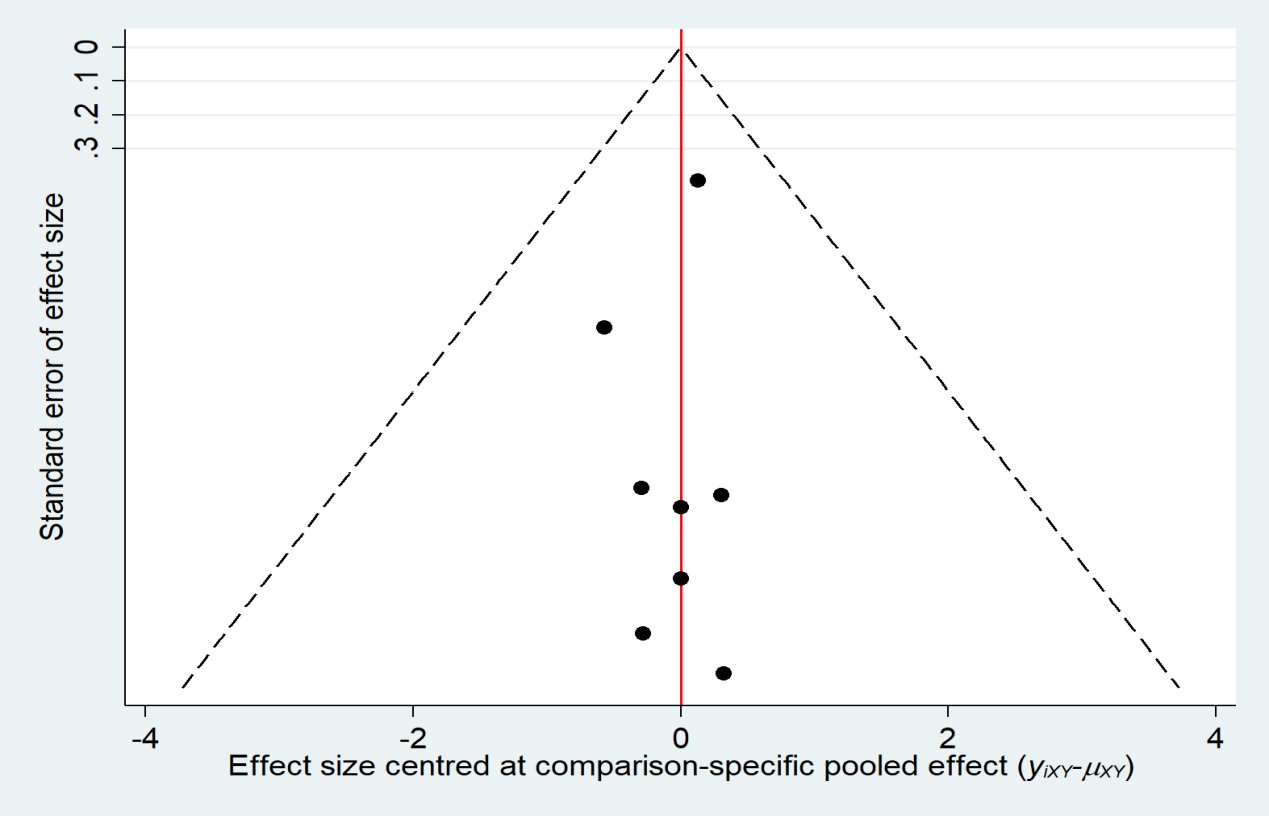


Figure S16. Funnel plot for strength.


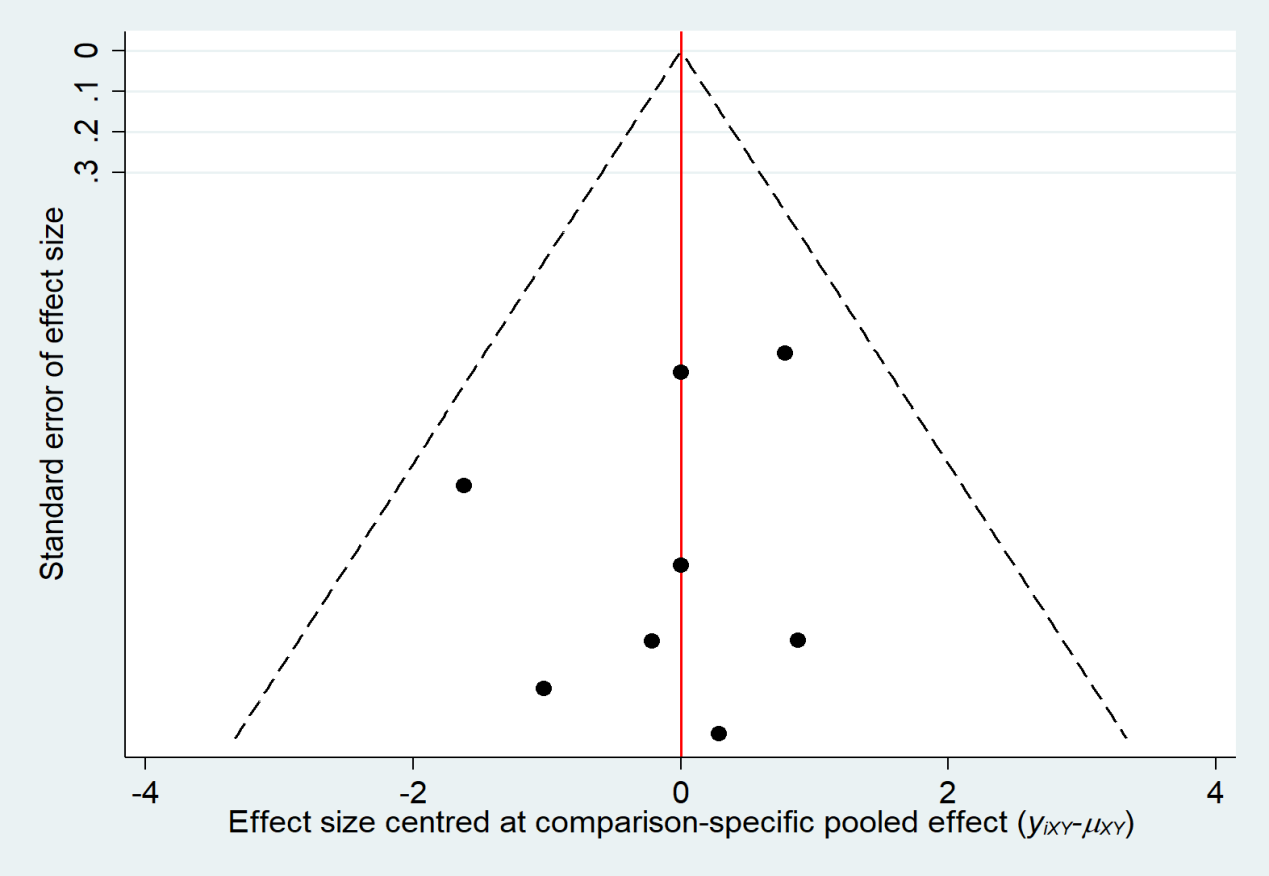


Figure S17. Funnel plot for activity of daily living.


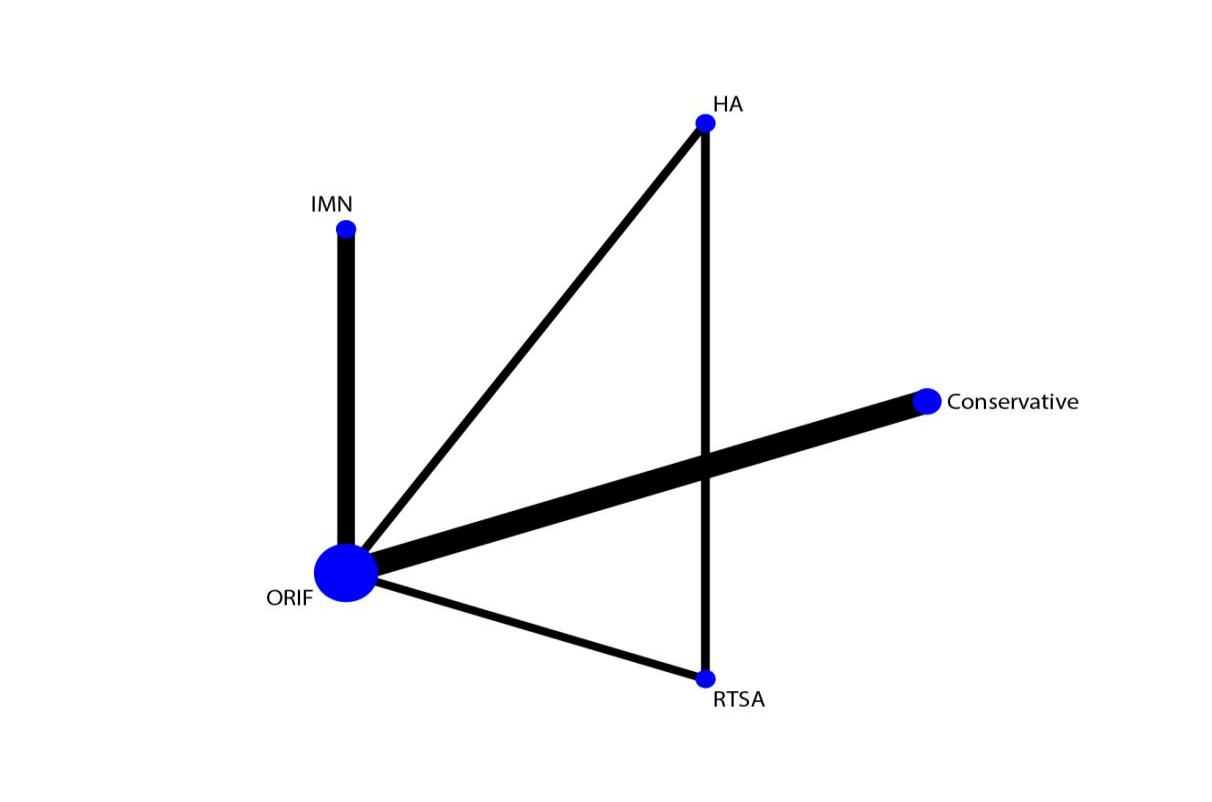


Figure S18. Network of comparisons for infection included in the analysis.


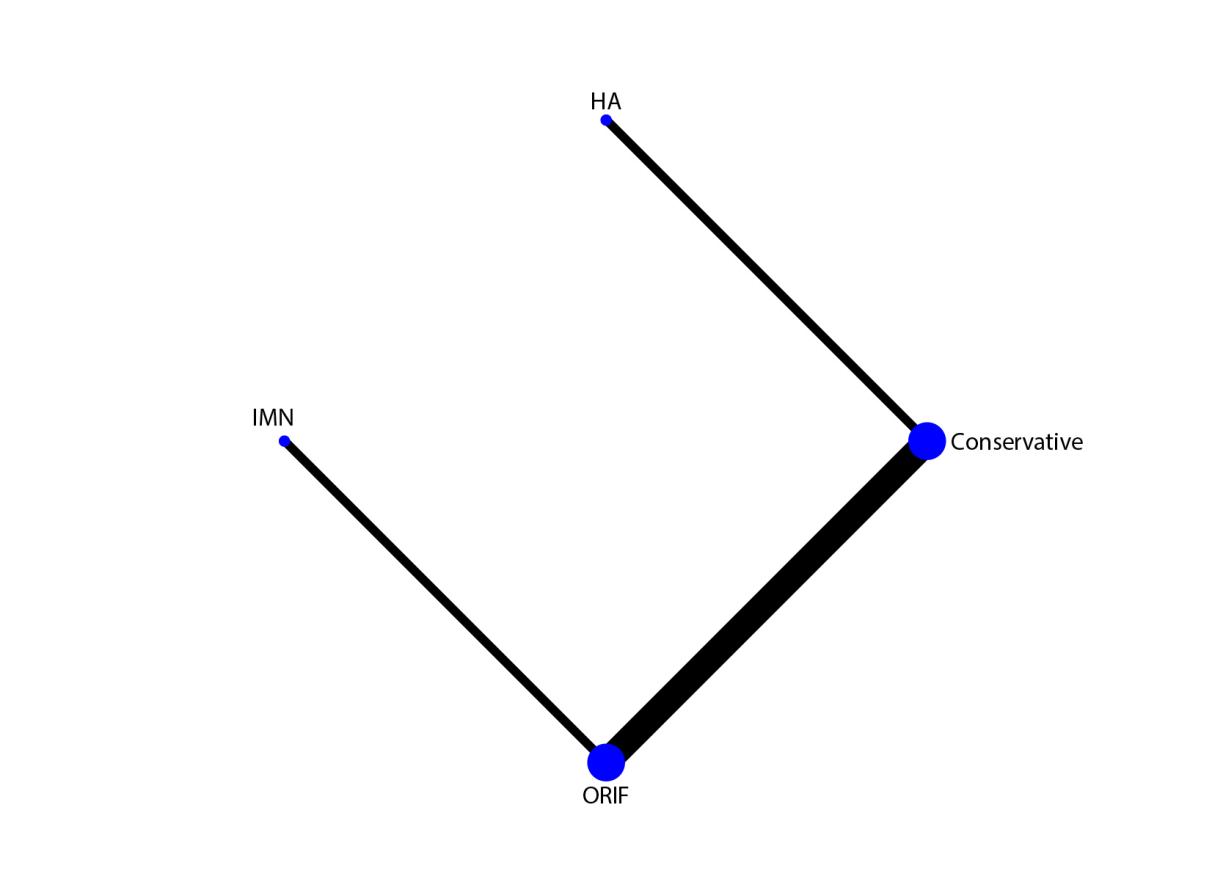


Figure S19. Network of comparisons for avascular necrosis included in the analysis.


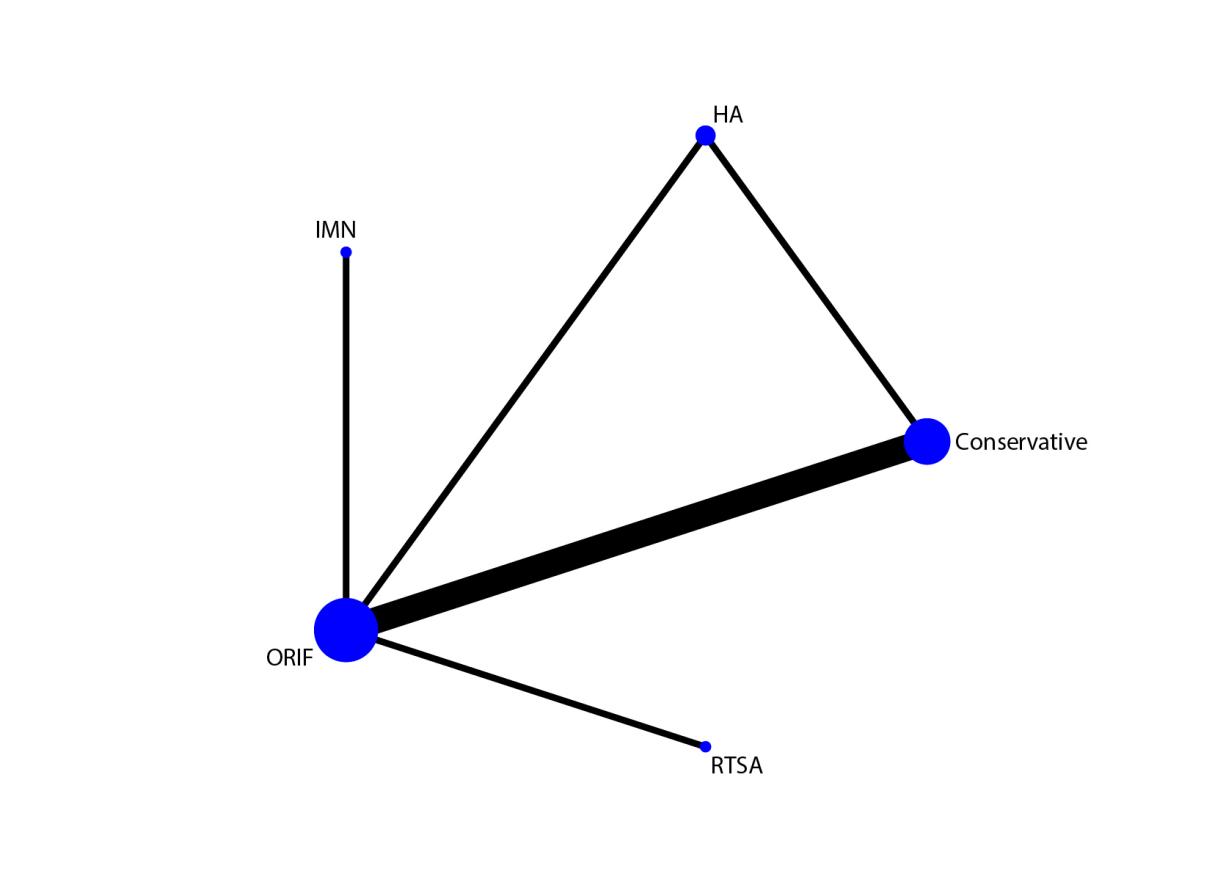


Figure S20. Network of comparisons for nonunion included in the analysis.


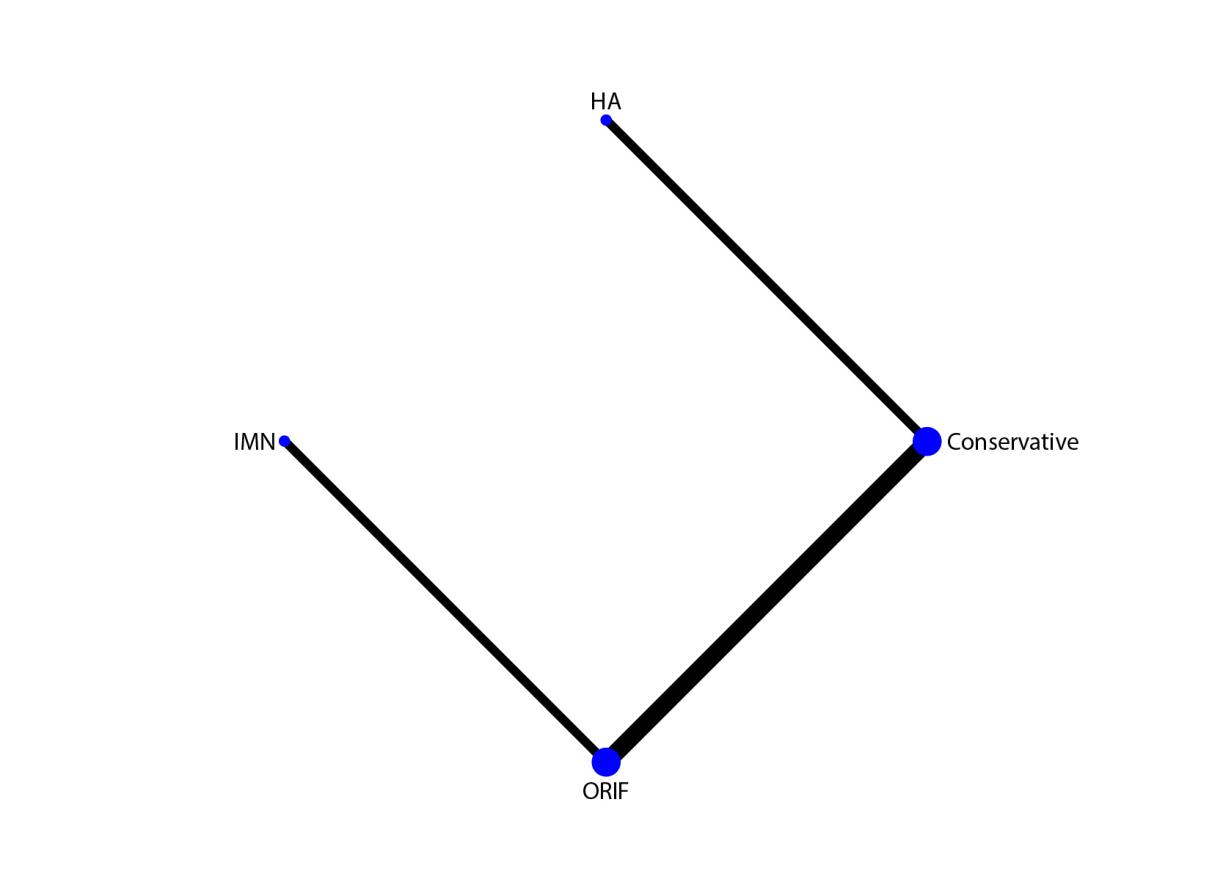


Figure S21. Network of comparisons for osteoarthritis included in the analysis.


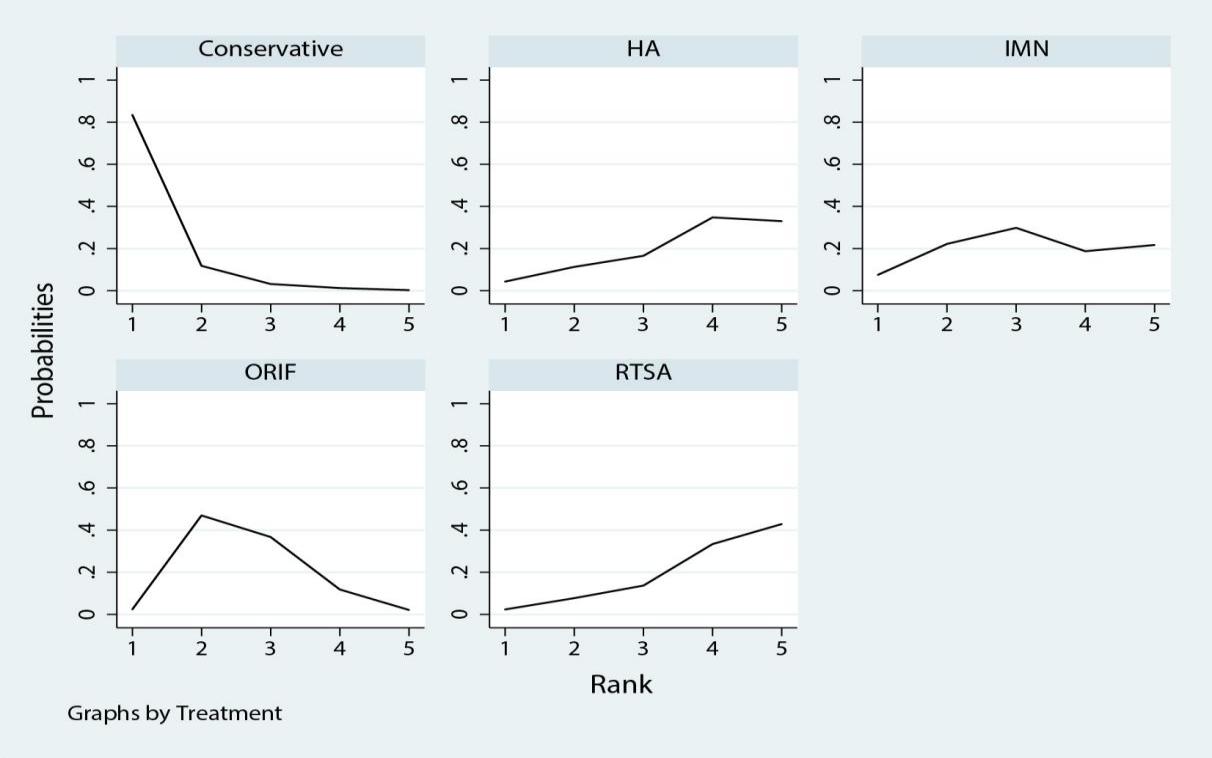


Figure S22. The SUCRA rank test for infection. Conservative: 94.2%; HA: 29.8%; IN: 43.8%; ORIF: 58.9%; RTSA: 23.3%


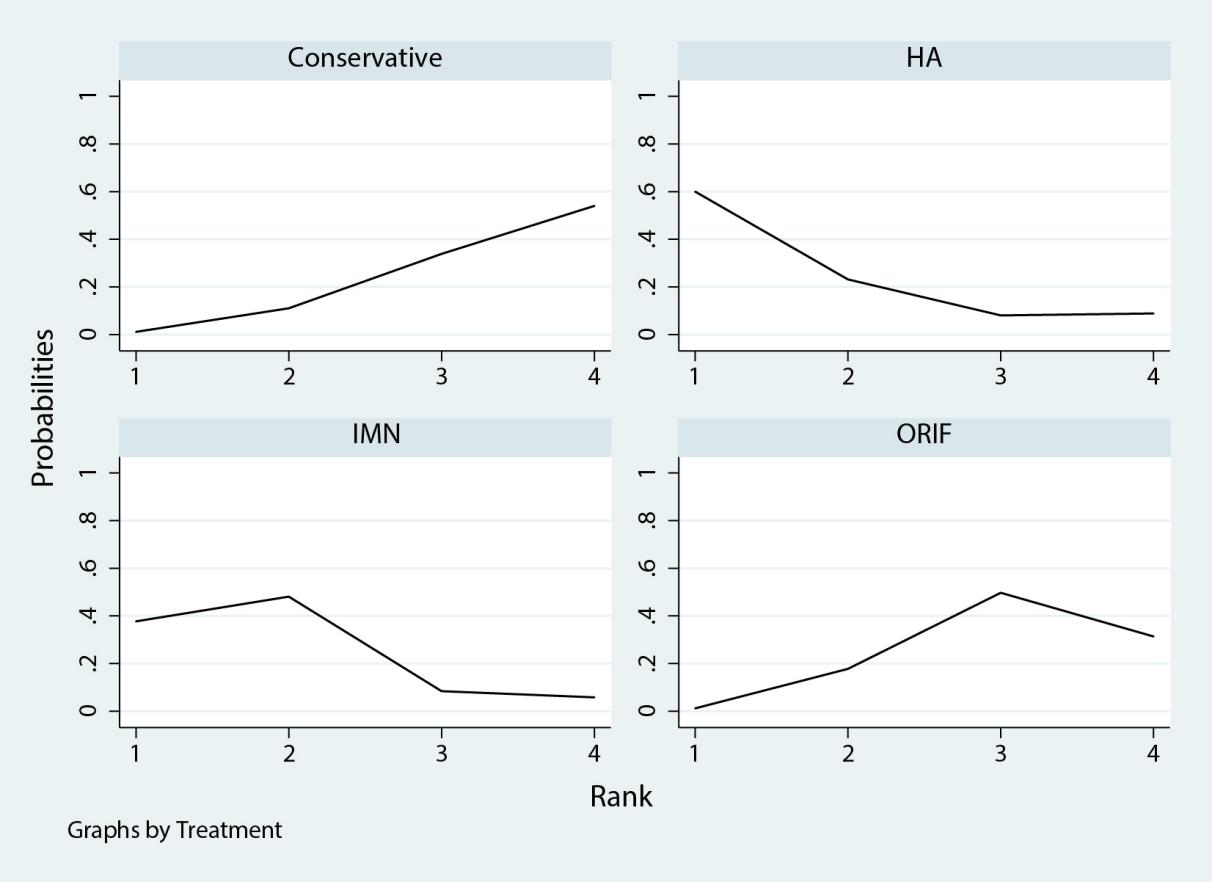


Figure S23. The SUCRA rank test for avascular necrosis. Conservative: 19.8%; HA: 78.1%; IN: 72.6%; ORIF: 29.6%


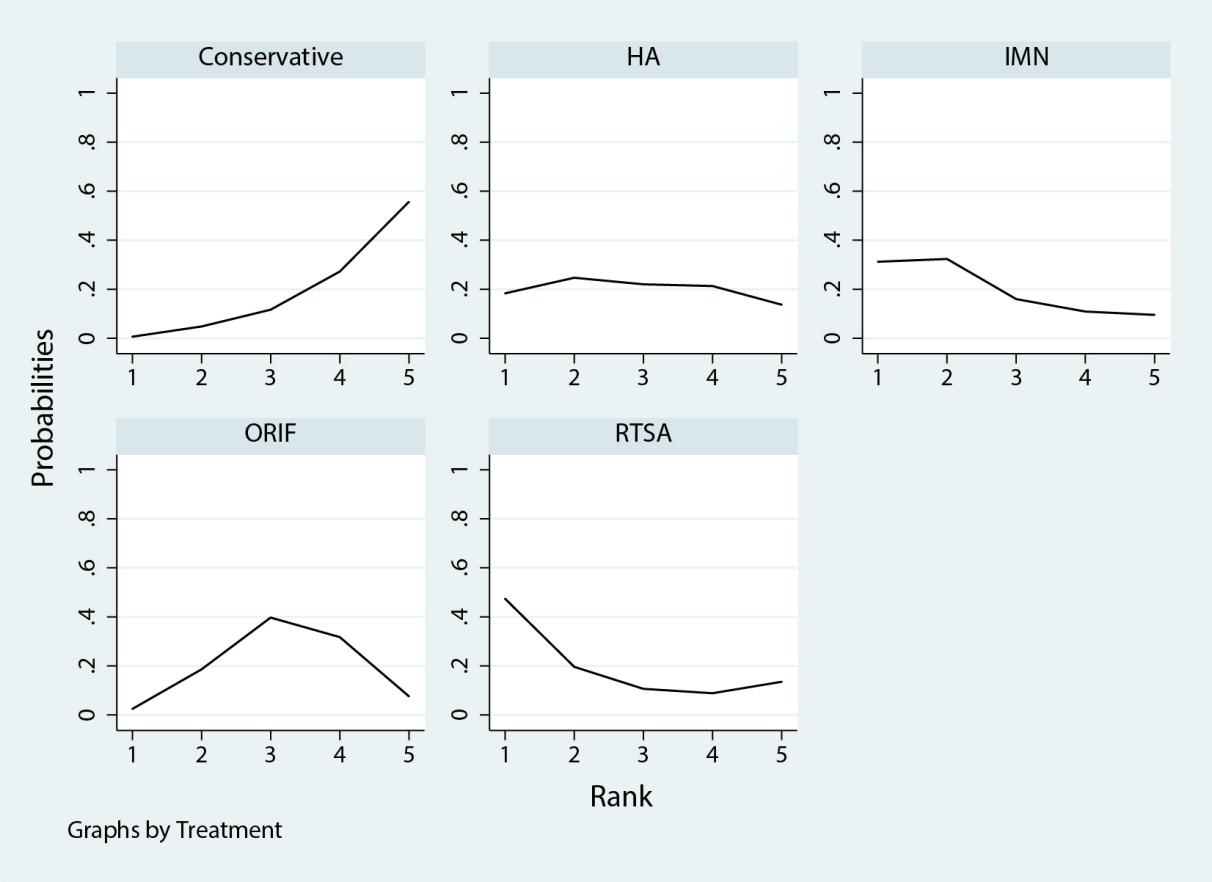


Figure S24. The SUCRA rank test for nonunion. Conservative: 16.9%; HA: 53.2%; IN: 66.2%; ORIF: 44.1%; RTSA: 69.6%


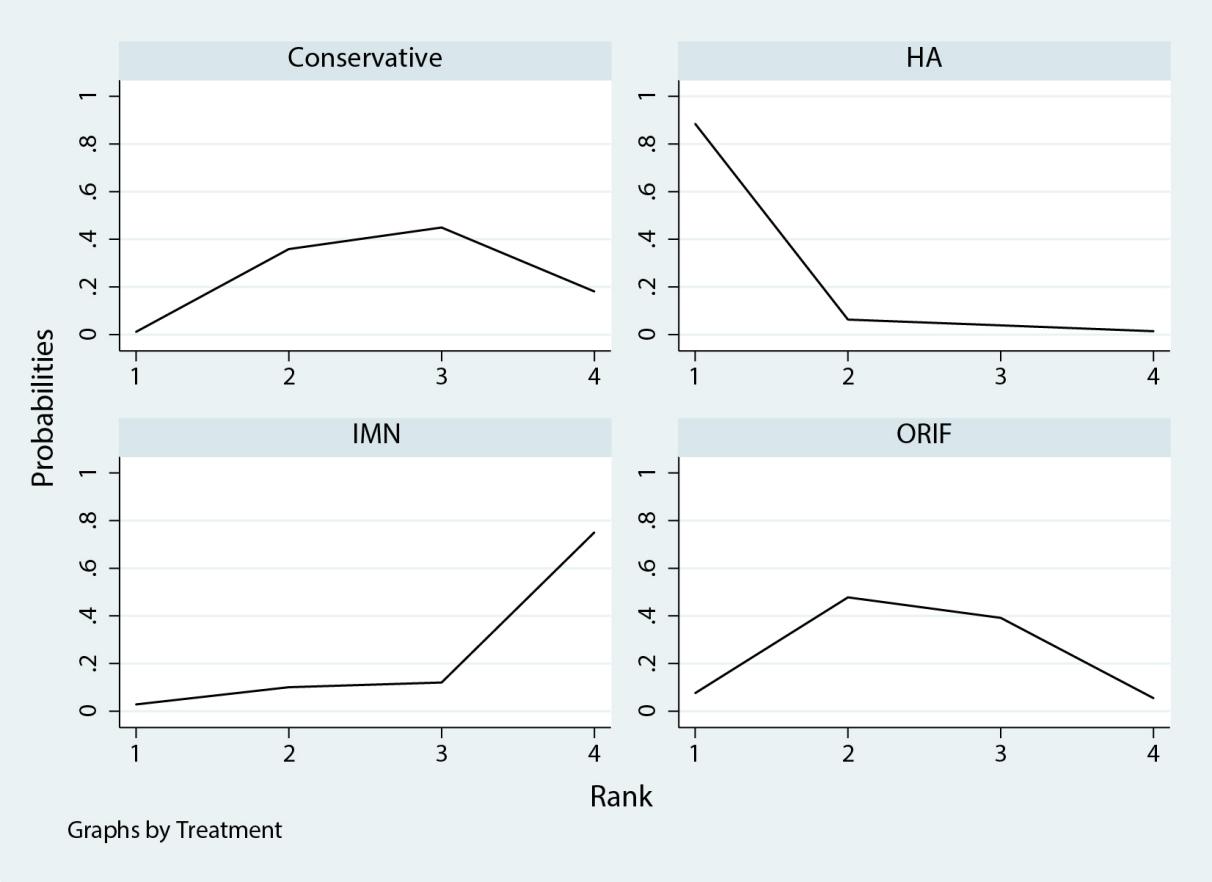


Figure S25. The SUCRA rank test for osteoarthritis. Conservative: 40.0%; HA: 93.9%; IN: 13.6%; ORIF: 52.5%


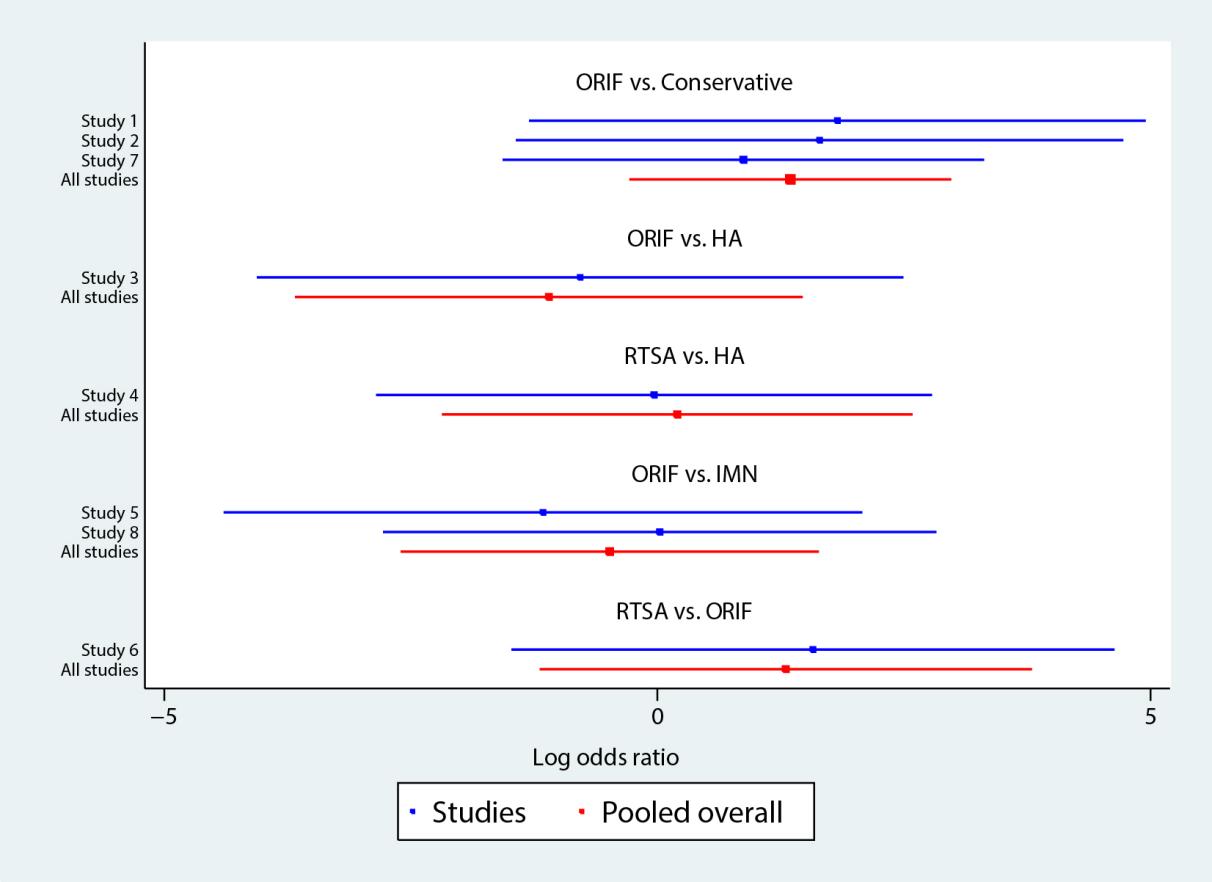


Figure S26. The pair-wise comparisons agents for infection.


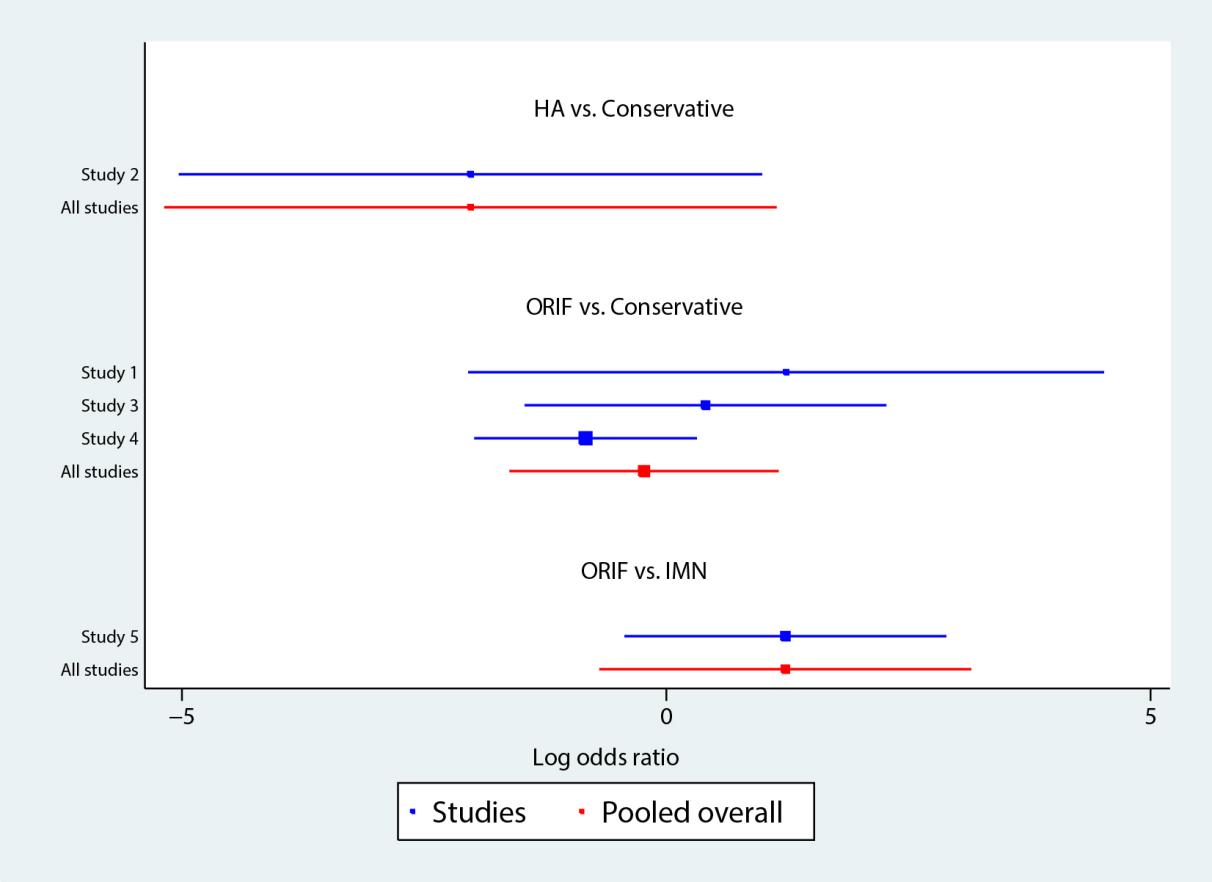


Figure S27. The pair-wise comparisons agents for avascular necrosis.


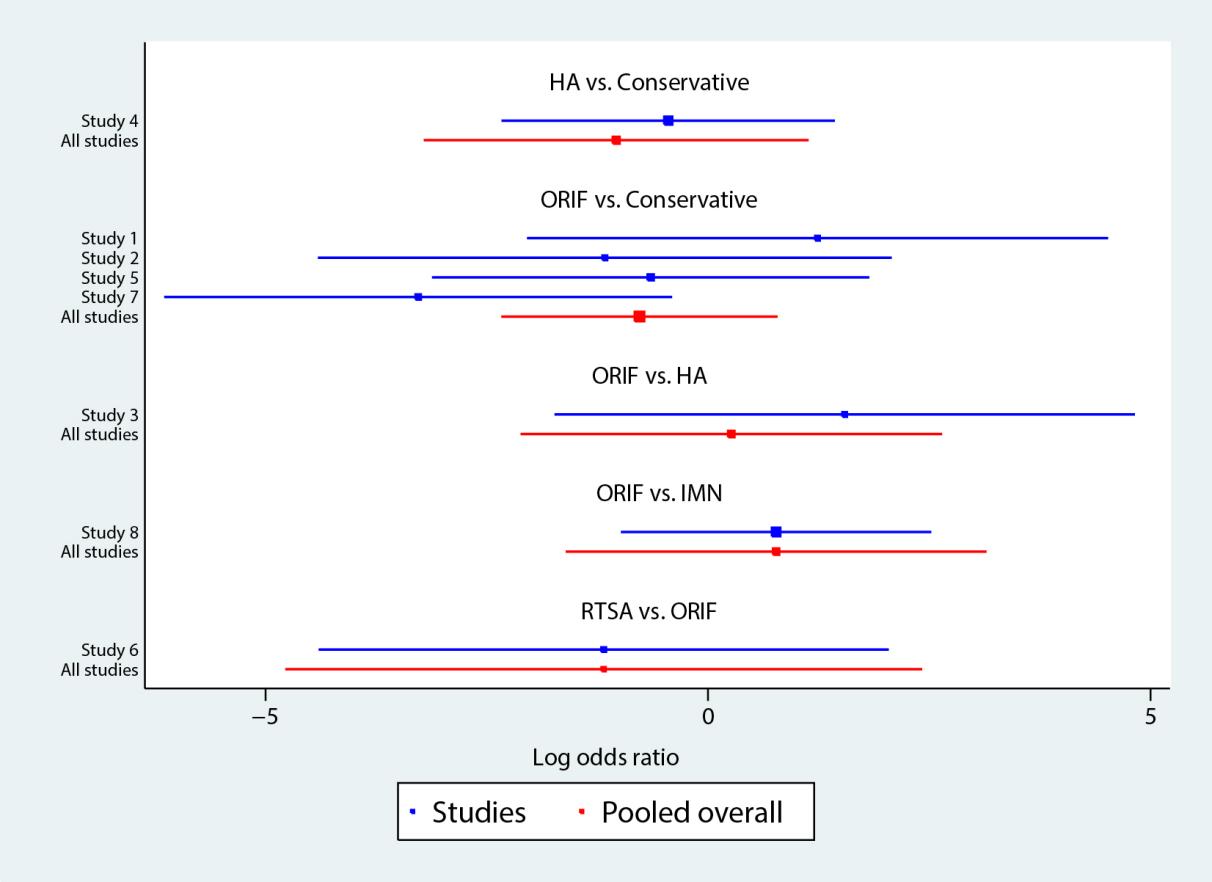


Figure S28. The pair-wise comparisons agents for nonunion.


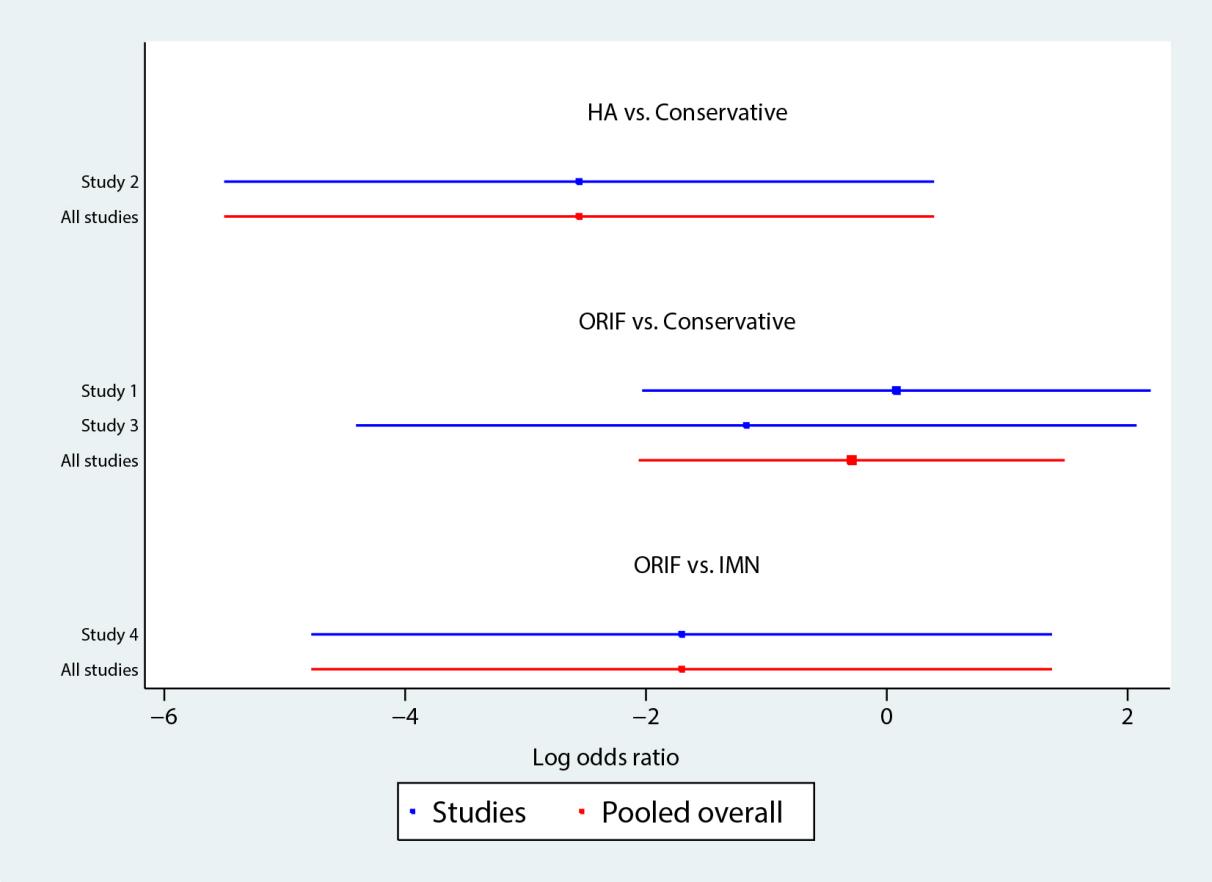


Figure S29. The pair-wise comparisons agents for osteoarthritis.


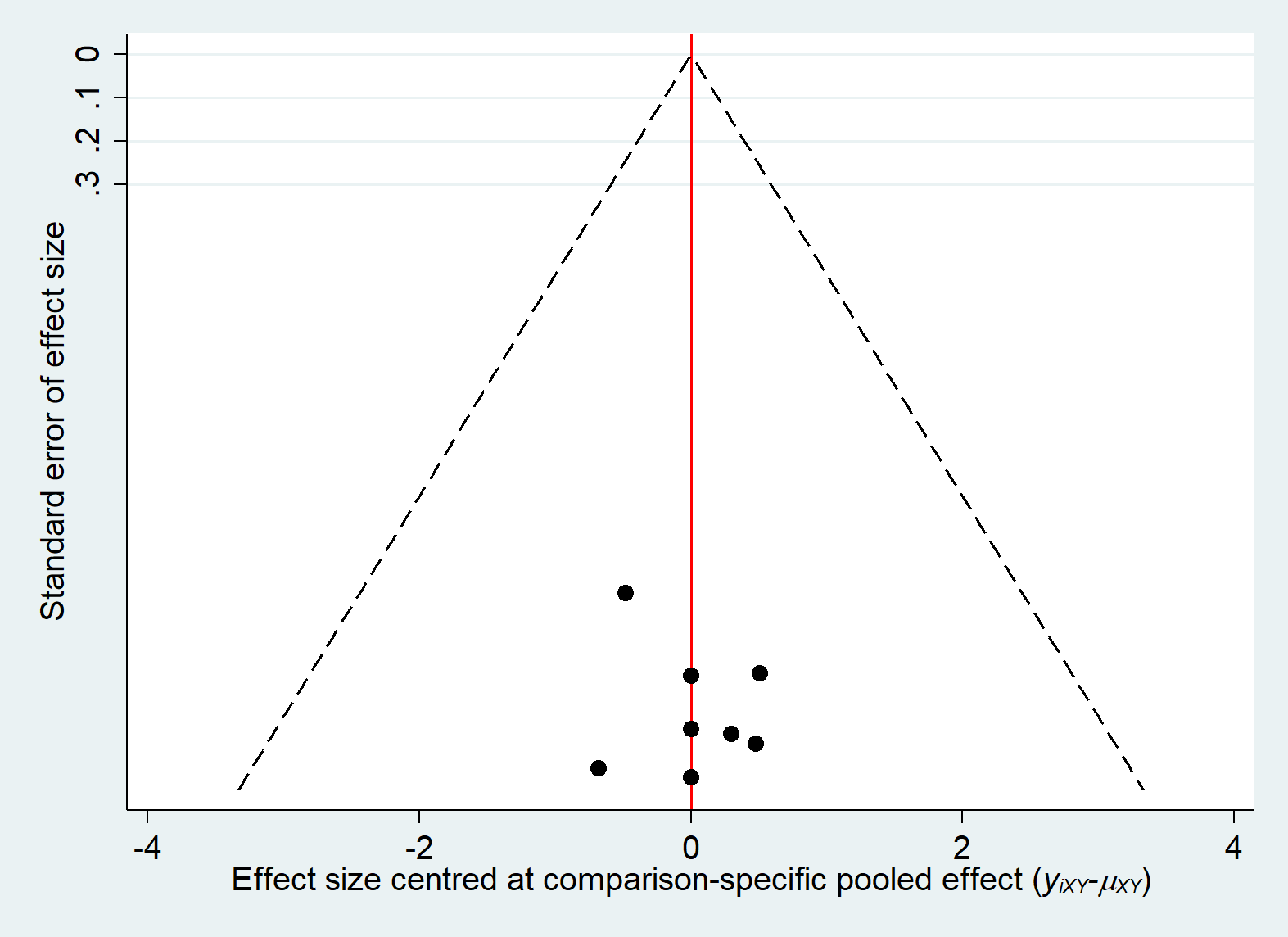


Figure S30. Funnel plot for infection.


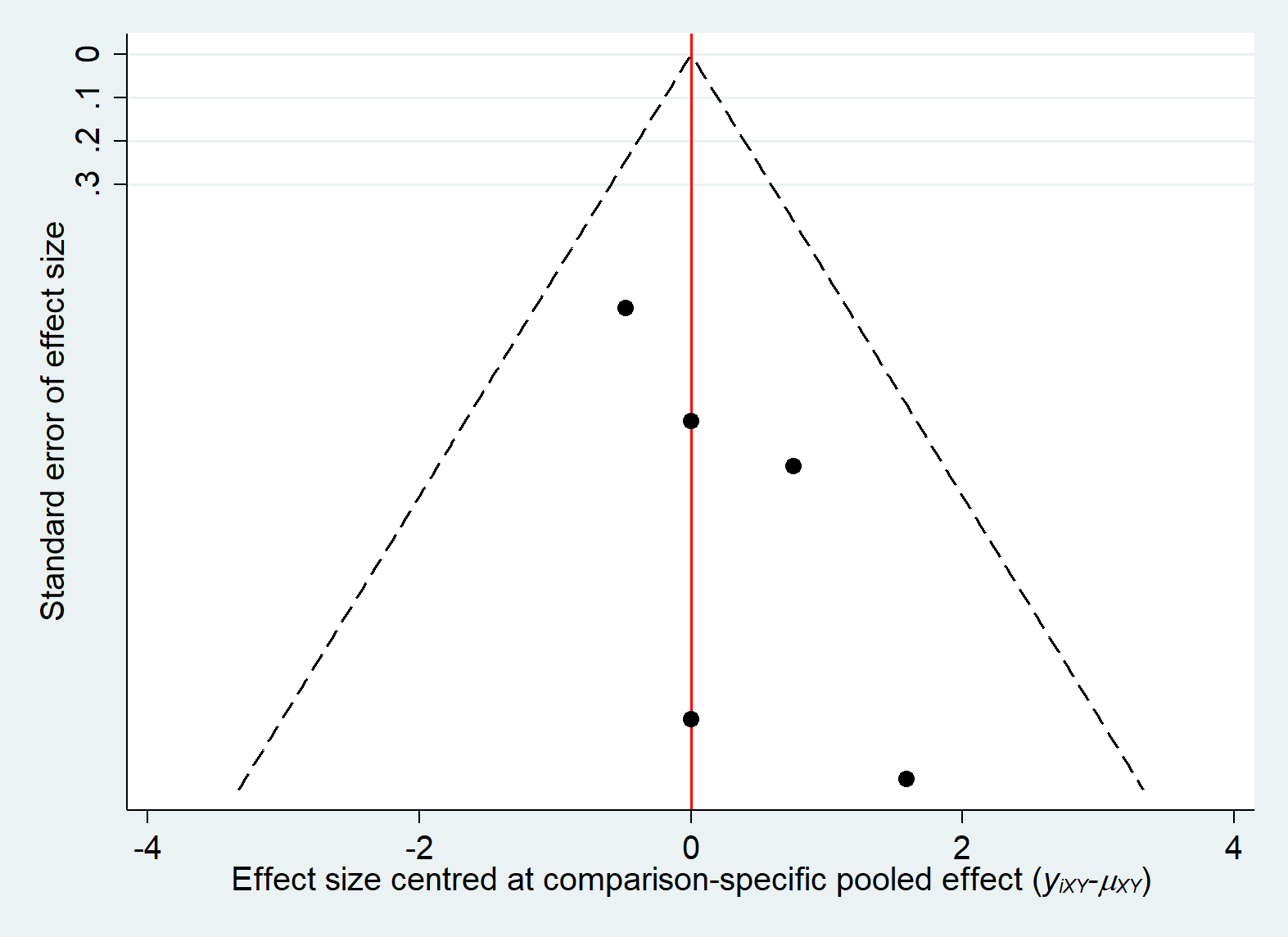


Figure S31. Funnel plot for avascular necrosis.


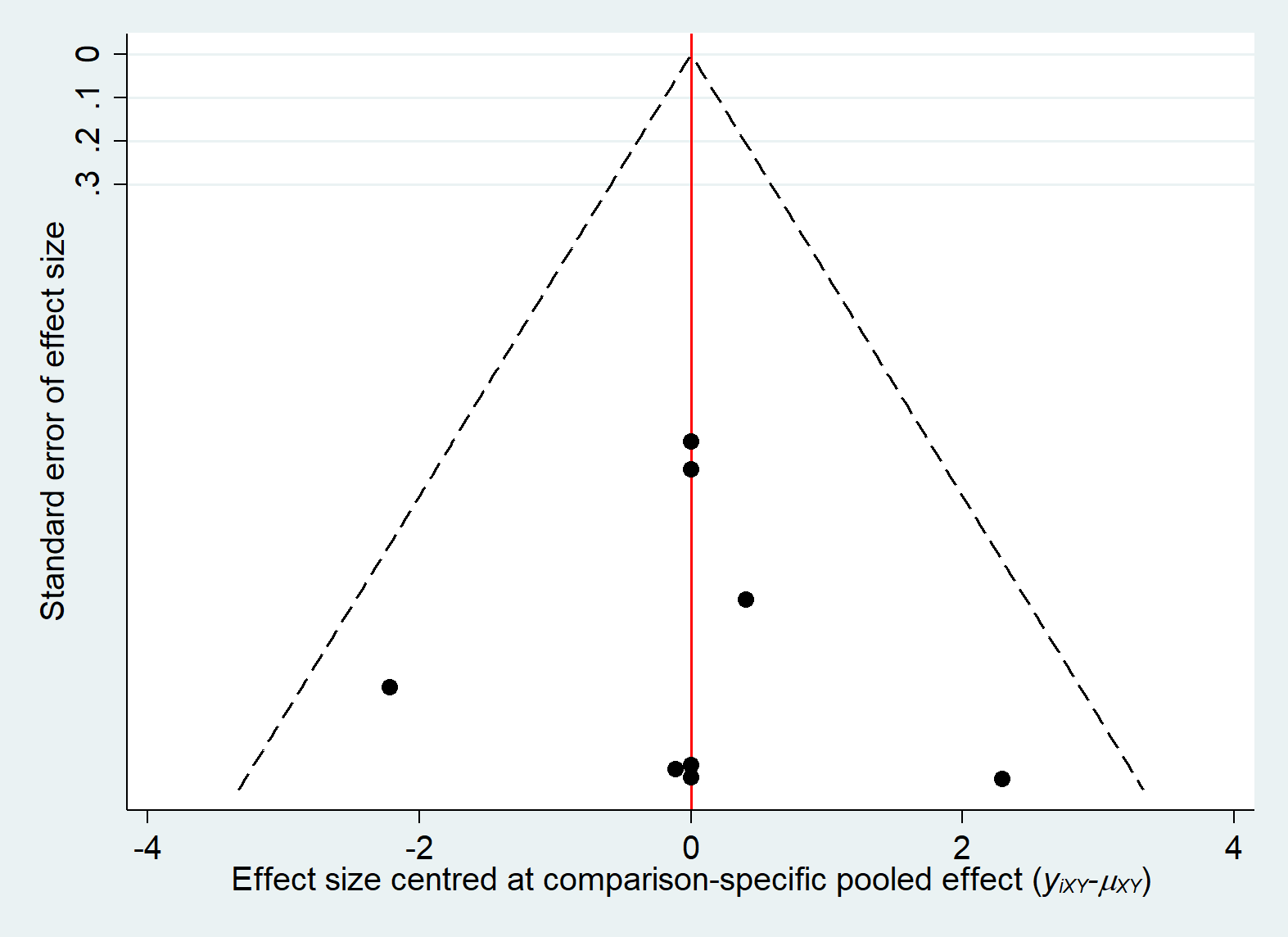


Figure S32. Funnel plot for nonunion.


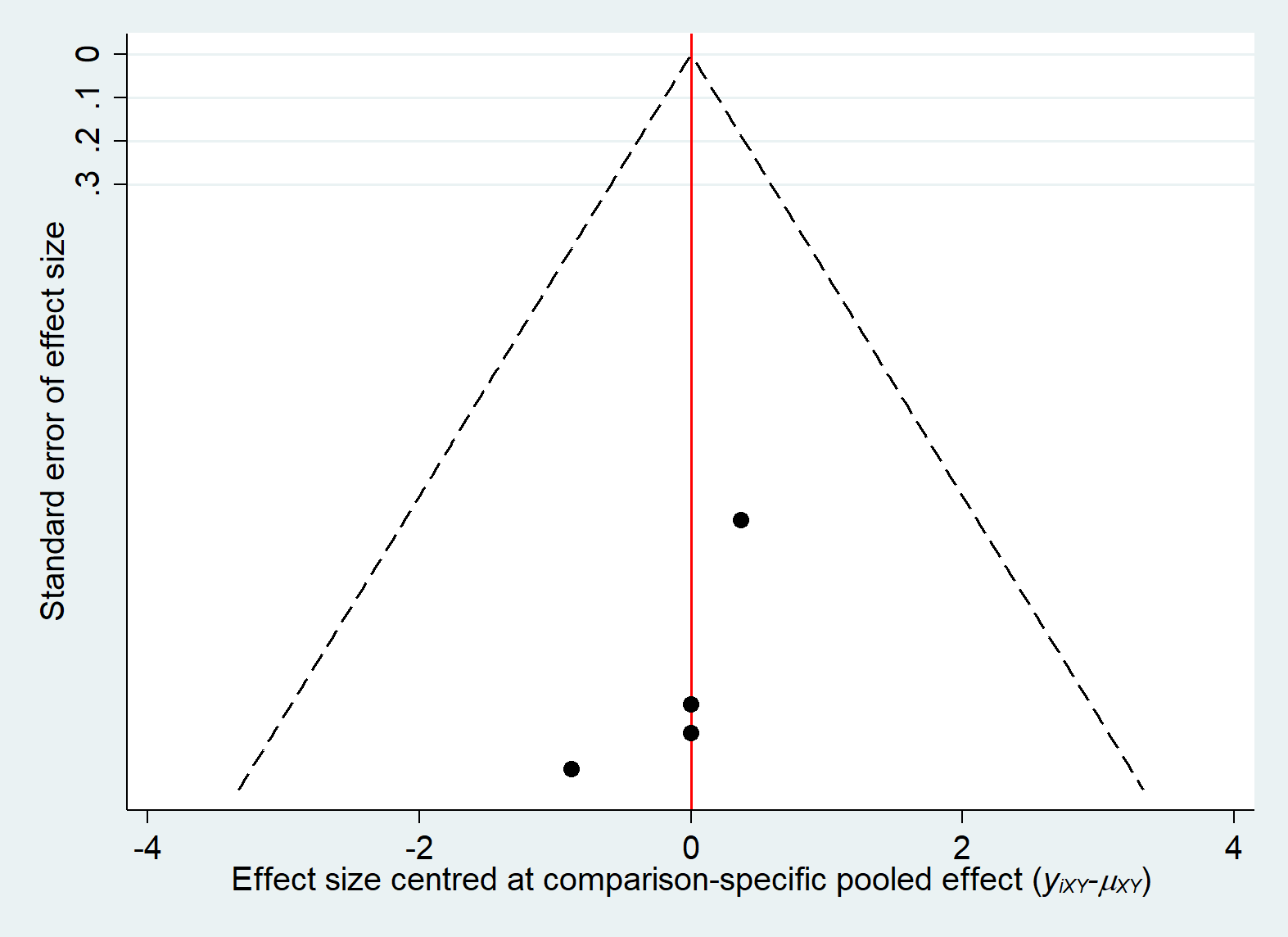


Figure S33. Funnel plot for osteoarthritis.
